# Chronophin regulates active vitamin B6 levels and transcriptomic features of glioblastoma cell lines cultured under non-adherent, serum-free conditions

**DOI:** 10.1186/s12885-018-4440-4

**Published:** 2018-05-03

**Authors:** Markus Schulze, Maria Hutterer, Anja Sabo, Sabine Hoja, Julia Lorenz, Tanja Rothhammer-Hampl, Christel Herold-Mende, Lucia Floßbach, Camelia Monoranu, Markus J. Riemenschneider

**Affiliations:** 1 Department of Neuropathology, Regensburg University Hospital, Franz-Josef-Strauss-Allee 11, 93053 Regensburg, Germany; 2 Experimental Neurosurgery, Department of Neurosurgery, University of Heidelberg, Heidelberg, Germany; 3 Department of Neuropathology, Institute of Pathology, University of Würzburg, Würzburg, Germany; 4 Wilhelm Sander-NeuroOncology Unit, Regensburg University Hospital, Regensburg, Germany

**Keywords:** Chronophin, Cofilin, Vitamin metabolism, Glioma

## Abstract

**Background:**

The phosphatase chronophin (CIN/PDXP) has been shown to be an important regulator of glioma cell migration and invasion. It has two known substrates: p-Ser3-cofilin, the phosphorylated form of the actin binding protein cofilin, and pyridoxal 5′-phosphate, the active form of vitamin B6. Phosphoregulation of cofilin, among other functions, plays an important role in cell migration, whereas active vitamin B6 is a cofactor for more than one hundred enzymatic reactions. The role of CIN has yet only been examined in glioblastoma cell line models derived under serum culture conditions.

**Results:**

We found that CIN is highly expressed in cells cultured under non-adherent, serum-free conditions that are thought to better mimic the in vivo situation. Furthermore, the substrates of CIN, p-Ser3-cofilin and active vitamin B6, were significantly reduced as compared to cell lines cultured in serum-containing medium. To further examine its molecular role we stably knocked down the CIN protein with two different shRNA hairpins in the glioblastoma cell lines NCH421k and NCH644. Both cell lines did not show any significant alterations in proliferation but expression of differentiation markers (such as GFAP or TUBB3) was increased in the knockdown cell lines. In addition, colony formation was significantly impaired in NCH644. Of note, in both cell lines CIN knockdown increased active vitamin B6 levels with vitamin B6 being known to be important for S-adenosylmethionine biosynthesis. Nevertheless, global histone and DNA methylation remained unaltered as was chemoresistance towards temozolomide. To further elucidate the role of phosphocofilin in glioblastoma cells we applied inhibitors for ROCK1/2 and LIMK1/2 to our model. LIMK- and ROCK-inhibitor treatment alone was not toxic for glioblastoma cells. However, it had profound, but antagonistic effects in NCH421k and NCH644 under chemotherapy.

**Conclusion:**

In non-adherent glioblastoma cell lines cultured in serum-free medium, chronophin knockdown induces phenotypic changes, e.g. in colony formation and transcription, but these are highly dependent on the cellular background. The same is true for phenotypes observed after treatment with inhibitors for kinases regulating cofilin phosphorylation (ROCKs and LIMKs). Targeting the cofilin phosphorylation pathway might therefore not be a straightforward therapeutic option in glioblastoma.

**Electronic supplementary material:**

The online version of this article (10.1186/s12885-018-4440-4) contains supplementary material, which is available to authorized users.

## Background

Gliomas, the most common type of primary brain tumor, diffusely infiltrate the adjacent brain tissue making complete surgical resection impossible [1]. The protein cofilin, a crucial regulator of actin dynamics, has been found to be a key regulator of migration and invasion in many types of cancer [2] including gliomas [3]. Active cofilin both supplies new barbed ends for actin polymerization [4] and promotes turnover of actin filaments [5]. The activity of cofilin is regulated by phosphorylation on a single serine residue, serine-3, which prevents its binding to actin filaments [6]. Upregulation of LIM-kinases that phosphorylate cofilin has been shown to lead to increased invasion in several types of cancer [7]. The pathway regulating cofilin phosphorylation is dysregulated in gliomas as compared to normal brain tissue in favor of an increased phosphorylation of cofilin [8].

It has been shown that proteins regulating cofilin phosphorylation, e.g. LIMKs and RHOC, are important regulators of pluripotency [9] and stemness in cancer cells [10]. This motivated us to examine the role of the cofilin phosphatase chronophin (CIN/PDXP) [11] in glioma cells cultured under non-adherent, serum-free conditions. These cells were derived under stem cell-permissive conditions [12] and are thought to better mimick the situation in the patient [13]. Of note, serum-cultured glioma cells deficient of CIN have a reduced capacity to grow in vivo [8] suggesting a loss of stemness after CIN reduction.

As CIN was initially described to dephosphorylate pyridoxal 5′-phosphate, the active form of vitamin B6 [14], we also examined the effects of CIN on this metabolite. Vitamin B6 is a cofactor in a multitude of enzymatic reactions [15] and regulates synthesis of S-adenosylmethionine (SAM). SAM levels and DNA-methylation as well as histone methylation are intimately linked with SAM being the main methyl donor for methylation reactions by DNA- and histone methylases [16]. Therefore, we hypothesized that CIN might be able to regulate stemness via alterations in cofilin phosphorylation or by altering the epigenome of the glioma initiating cells (by inducing global hypermethylation). Indeed, a subset of preferentially proneural and *IDH*-mutated gliomas shows a global hypermethylator phenotype [17]. However, as not all gliomas that show global hypermethylation carry *IDH*-mutations, CIN downregulation might be an alternative mechanism inducing a global shift in methylation patterns [18]. It should be noted that although CIN is downregulated in the tumor bulk in glioblastomas [8] tumor initiating cells may be a rare population in glioblastomas [19]. The estimated frequencies vary widely from well below 1% [19] up to ~ 10–30% [13, 19] depending on the assay used for quantification. Therefore a role of CIN in regulation of cell growth or stemness does not contradict its downregulation in the tumor bulk.

Both regulation of cofilin phosphorylation [20] as well as vitamin B6 [21] have been implicated in modulation of chemoresistance. Inhibitors for LIM-Kinases and ROCK (Rho-associated protein kinase), regulating cofilin phosphorylation [4], are readily available. We therefore also explored the potential to modulate chemoresistance in glioblastoma stem-like cells by applying these inhibitors.

## Methods

### Cell culture

We used five adherent glioblastoma cell lines cultured in serum: T98G, TP365, U87, U251, A172. T98G (#CRL-1690), U87 (#HTB-14) and A172 (#CRL-1620) were from ATCC (Manassas, VA, USA). TP365 and U251 were kindly provided by Prof. V. P. Collins, Cambridge, UK. Identity of all cell lines was confirmed by short tandem repeat analysis. The cells were cultured in complete medium consisting of high glucose (4.5 g/L) Dulbecco’s modified Eagle’s medium with stable glutamine (PAN Biotech, Aidenbach, Germany) supplemented with 100 U/ml penicillin, 100 μg/ml streptomycin (GIBCO) and 10% fetal bovine serum (PAN Biotech). Five previously characterized [12] human primary stem-like glioblastoma cell lines (NCH1425, NCH421k, NCH465, NCH601, NCH644, all supplied by Prof. Christel Herold-Mende, Heidelberg, Germany) were cultured in DMEM/Ham’s F-12 medium with stable glutamine supplemented with 100 U/ml penicillin, 100 μg/ml streptomycin, 20% BIT admixture supplement (Pelo Biotech, Planegg, Germany), 20 ng/ml epidermal growth factor (EGF) (ReliaTech, Wolfenbuettel, Germany) and 20 ng/ml basic fibroblast growth factor 2 (bFGF2) (ReliaTech). Cells were handled under aseptic conditions and grown at 37 °C and 5% CO_2_. All cells were tested for mycoplasma contamination.

### Establishment of stable shRNA cell lines

Plasmids coding for different validated CIN targeting shRNAs in a pLKO.1-puro backbone (TRCN0000050044, TRCN0000050046) and the SHC002 plasmid, a control shRNA (Sigma-Aldrich, St. Louis, USA), were transfected in HEK293T cells with lipofectamine 2000 (Life Technologies) together with a third generation lentiviral packaging system (Addgene, Cambridge, MA, USA) and the p-advantage vector (Promega, Fitchburg, WI, USA). 72 h and 96 h after transfection, supernatants were harvested and remaining cells were removed from the solution by centrifugation for 5 min at 100 *x g*. To remove any serum remnant from the HEK293T cell medium, which would induce differentiation of the stem-like cells, we purified the lentiviral particles twice with PEGit (System Biosciences, Palo Alto, CA, USA) according to the manufacturer’s instructions. Briefly, for one round of purification, 4 parts supernatant were mixed with 1 part PEGit, and the solution was incubated O/N at 4 °C. Viral particles were collected by centrifugation at 1500 *x g*, for 30 min at 4 °C. The supernatant was discarded and the remaining pellet and liquid recentrifuged for 5 min and 1500 *x g* at 4 °C. After complete removal of the supernatant, the remaining pellet was dissolved in 4.5 ml DMEM/F12 for further purification or transduction of stem-like cells. We determined the concentration of the lentiviral particles with a p24 ELISA kit (Cellbiolabs, San Diego, CA, USA) and infected the cells at an MOI of 5. Two days after transduction 1 μg/ml puromycin (Sigma-Aldrich, St. Louis, USA) was added to the culture medium to select for shRNA expressing cells. After two weeks of selection we obtained robustly proliferating cell cultures. For simplicity, TRCN0000050044 is hereafter referred to as CIN shRNA #1, TRCN0000050046 as CIN shRNA #2 and SHC002 as CTRL.

### Proliferation, chemosensitivity and cell viability assay

For proliferation assays, 2000 stably transduced NCH421k and NCH644 cells were seeded in five separate 96-well plates in a final volume of 100 μl. Every day, 10 μl resazurin (R&D Systems, Minneapolis, MN, USA) were added to one plate, incubation was performed for 3 h at 37 °C and 5% CO2 and fluorescence intensity was measured at in a FLUOstar Omega microplate reader at Ex544nm/Em590nm (BMG Labtech, Ortenberg, Germany). After background (medium w/o cells plus resazurin) substraction the values were expressed as fold of the intensity at day 1. The chemotherapeutic agent temozolomide (Sigma-Aldrich) was dissolved in DMSO at concentrations of 200 mM. The ROCK-inhibitors Y-27632 (Sigma-Aldrich) and fasudil (Tocris Bioscience, Bristol, UK) were dissolved in sterile ultrapure water (Carl-Roth, Karlsruhe, Germany) at a concentration of 10 mM. The LIMK-inhibitor LIMKi3 (Tocris Bioscience) was dissolved in DMSO at a concentration of 10 mM. All reagents were thawed three times at maximum. For chemosensitivity assays, 1000 NCH644 or NCH421k cells were seeded per well on a 96-well plate in stem cell medium. The cells were treated with 10 serial dilutions of temozolomide ranging from final concentrations of 1000 to 0.01 μM. Then, Y-27632, fasudil or LIMKi3 were added in a final concentration of 10 μM (in a final volume of 200 μl), a concentration chosen based on literature reports [22–24]. DMSO and water served as a control. The plates were incubated for 96 h, 20 μl of resazurin were added and measurement of resazurin fluorescence intensity was performed as has been described above. For the chemosensitivity assays of shRNA cells the protocol was performed without the inhibitor treatement.

### (PhosTag) western blotting

For western blot cells were washed in DPBS supplemented with 1% BSA and lysed in 150 μl of RIPA lysis buffer with added phosphatase and protease inhibitor cocktail (Roche, Basel, Switzerland) and kept on ice. The lysates were mixed with Laemmli buffer, denatured at 90 °C for 5 min. DNA was sheared with a 20G × 1.5″ needle and the samples were run on 8–15% SDS-PAGE gels depending on the size of the analyzed protein. MagicMark™ Western Protein Standard (Life Technologies) or Color Prestained Protein Standard, Broad Range (NEB, Ipswich, MA, USA) were used as a molecular weight marker. Gels were run at a constant voltage of 80 V for 30 min (stacking gel) followed by 140 V for 60–70 min (separating gel), dependent on the polyacrylamide concentration of the gels. For separation of cofilin and phosphocofilin PhosTag was added to the gels as has been described previously [8]. Protein was blotted from the SDS-PAGE gels on 0.45 μm nitrocellulose membranes (Bio-Rad, Munich, Germany) with a semi-dry Fastblot B44 (Biometra, Goettingen, Germany). Afterwards, the membrane was blocked using 5% non-fat dry milk for 1 h followed by incubation in primary antibody over night at 4 °C. The primary antibodies were diluted 1:10,000 (Tubulin, mouse antibody [Clone DM1A], Sigma-Aldrich) or 1:1000 for CIN (rabbit antibody [clone C85E3], Cell Signaling Technologies, Danvers, CO, USA), p-Ser3-cofilin (rabbit antibody [clone 77G2], Cell Signaling Technologies) and cofilin (rabbit antibody [clone D3F9], Cell Signaling Technologies). The next day, the membrane was washed three times in TBS-T for 2 min and then the primary antibody was detected by anti-rabbit or anti-mouse IgG linked to horseradish peroxidase (Santa Cruz, Dallas, Texas, USA) diluted 1:10,000 in a solution containing 5% non-fat dry milk for 1.5 h at RT. Picoluminescence substrate **(**Thermo Fisher Scientific, Waltham, Massachusetts, USA) was used for development on a LAS4000 imaging system (GE healthcare, Munich, Germany). Quantification was performed with ImageQuant TL ver. 7.0 (GE healthcare).

### RNA / DNA isolation and cDNA synthesis

RNA and DNA were isolated with the RNA/DNA Allprep kit (Qiagen, Hilden, Germany) according to the manufacturer’s instructions. cDNA synthesis was performed from 1 μg total RNA using random hexamer primers (Gene Link, Hawthorne, NY, USA) and the SuperScript™ II Reverse Transcriptase (Life Technologies). RNA from normal human astrocytes was commercially available (ScienCell Research laboratories, Corte Del CedroCarlsbad, CA).

### Real-time (RT-)PCR

Real-time RT-PCR was performed with the SensiFAST™ SYBR Hi-Rox Kit (Bioline, London, UK) on the StepOnePlus™ cycler (Life Technologies). Relative expression values were calculated with the ΔΔC_T_ (analysis of relative gene expression) method [25] using *ARF1* as the reference transcript. Primers used for CIN were 5′-CTGGAGACCGACATCCTCTTT (forward) and 5′-TTCTAGGCGGGAGACTCCTG (reverse), for ARF1 5′-GACCACGATCCTCTACAAGC (forward) and 5′-TCCCACACAGTGAAGCTGATG (reverse), for c-Myc 5′-TCGGATTCTCTGCTCTCCTC (forward) and 5′- TCATCTTCTTGTTCCTCCTCAGA (reverse) for *NES* 5′-ATCGCTCAGGTCCTGGAA (forward) and 5′-AAGCTGAGGGAAGTCTTGGA (reverse) and for GFAP 5′-TGAAGCCGAAGAGTGGTACC (forward) and 5′-GGTAGTCGTTGGCTTCGTG (reverse). TUBB3 primers were described by others [26]. Analysis of the *CDKN2A* deletion status was performed with four different primer sets targeting the *CDKN2A* locus as has been described by others [27, 28] with the real-time PCR conditions as described above and primers for human *B2M* as reference [29] as well as a commercially available human genomic DNA (Roche) as control.

### Isolation of metabolites and measurement of active vitamin B6 levels

Metabolites were isolated as has been described by others [30] with the exception that we used DPBS supplemented with 0.9% NaCl and 1% fraction V BSA for quenching, which was performed at 4 °C. These changes were necessary because extensive cell rupture occurred without BSA and by incubation on ice. It should be noted that vitamin B6 binds to BSA, albeit with lower affinity than to human serum albumin [31]. However, all BSA containing media were carefully removed before metabolite extraction and the amount of BSA used for quenching and washing was identical for every cell line. Therefore, the interference of BSA with the measured vitamin B6 level is identical across samples. The extracts were dried in a Savant SpeedVac-concentrator and finally resuspended in water to equal 20,000 cells/μl H_2_O. Active vitamin B6 levels were measured with an enzymatic kit (Buehlmann Laboratories, Schoenenbuch, Switzerland) according to the manufacturer’s instructions. The kit was validated to yield comparable results to HPLC by the manufacturer and enzymatic methods have been shown to yield comparable results to HPLC in general [32]. Briefly, substrate was added to each well of a 96-well plate and an equal volume of 1:40 diluted sample was added. Afterwards, apoenzyme was added, and the plate was shaken for 15 s at 400 rpm. The mixture was incubated for 30 min at 37 °C, enzyme was added and the plate was shaken for 15 s. Then, another incubation was performed for 15 min. at 37 °C and the OD546 was measured.

### Bisulfite treatment and LINE-1 PCR

Bisulfite conversion of 1 μg genomic DNA was performed with the EpiTect bisulfite kit (Qiagen, Hilden, Germany) according to the manufacturer’s instructions. Global DNA-methylation levels were estimated via LINE-1 bisulfite PCR [33]. Amplification of LINE-1 elements was performed with HotStarTaq DNA polymerase (initial denaturation at 95 °C for 15 min, 35 cycles of 94 °C for 90s, 50 °C for 60s, 72 °C for 60s and a final elongation step at 72 °C for 10 min). Sequencing was performed on a Pyromark Q24 instrument (Qiagen) following standard protocols.

### Analysis of histone methylation

Cells were counted and ~ 1.5 × 10^5^ cells were washed and resuspended in DPBS containing 1% BSA, fixed with 4% PFA for 15 min and collected by centrifugation at 500 *x g* for 5 min. Afterwards, the cells were blocked and permeabilized in DPBS supplemented with 0.5% TritonX-100 and 5% normal goat serum (PAN Biotech). After two washing steps with DPBS supplemented with 0.5% TritonX-100, the cells were stained with 1 μg Histone-K27me3 antibody (rabbit polyclonal, Millipore, Billerica, MA, USA) and a rabbit isotype control (rabbit clone [DA1E], Cell Signalling Technologies) for 90 min. in 100 μl DPBS supplemented with 0.5% TritonX-100 and 1% normal goat serum. Afterwards, cells were washed twice, stained with a 1:200 diluted Alexa-Fluor 488 conjugated anti-rabbit secondary antibody (Thermo Fisher Scientific) for 90 min, counterstained with 1 μg/ml DAPI for 10 min and washed again twice. Finally, the cells were resuspended in DPBS and analyzed on a BD FACS CantoII flow cytometer. Here, DAPI positive single cells were gated and analyzed for their fluorescence signal.

### Colony formation assay

Colony formation was assessed with the reagents and procedures as described previously [12] with the following modifications. We increased the cell number to 3000 cells per well, but reduced the incubation time to two weeks. For inhibitor treatment assays, Y-27632 was added to the collagen solution as well as to the feeding medium at the concentrations indicated. Water was used as a control.

### Library preparation for RNA-Seq

Libraries for next-generation sequencing were prepared from 600 ng total RNA with the TrueSeq RNA library preparation kit v2 as has been described previously [34]. Illumina deep sequencing was performed at a genomics core facility: Center of Excellence for Fluorescent Bioanalytics (KFB, University of Regensburg, Germany) on a HiSeq1000 instrument.

### NGS data analysis

Analysis of NGS data was performed using the Genomatix software (Genomatix, Munich, Germany). First, the .fastq files were mapped to the human genome assembly GRCh38 (annotation based on ElDorado 6–2015) using the Genomatix Mining Station Mapper v3.7.6.3 allowing one mismatch. All unique hits were further processed using the Genomatix Genome Analyzer v3.51106 which was used to create count tables for all samples. Reads were counted locus-based, i.e. for unions of exons of genes. All further analyzes were performed with the free software R v3.1.1, Bioconductor v3.0 [35] and the package DESeq2 v1.6.3 [36]. Gene set enrichment analysis [37] was performed with the ssGSEA module v7 [38] with RPKM (reads per kilobase of exon model per million mapped reads) values [39]. Subtype prediction was performed with the ssGSEA module and the gene sets proposed by ref. [40].

### Data analysis

All analyzes were performed with GraphPad Prism 5.0 and R ver. 3.1.1. If not otherwise indicated, two-sided t-tests were used for statistical analysis of two groups. For three or more groups, a one-way ANOVA followed by Dunnett’s multiple comparison test was applied. A result was accepted as significant if p was < 0.05 and significant differences were indicated where present (* *p* < 0.05, ** *p* < 0.01 and *** *p* < 0.001). For the determination of IC_50_ values, the concentration of temozolomide used was log-transformed, the fluorescence values were normalized and a fit with variable slope was performed.

## Results

### Proteins regulating cofilin phosphorylation are strongly downregulated in stem-like glioma cells

**Fig. 1:**
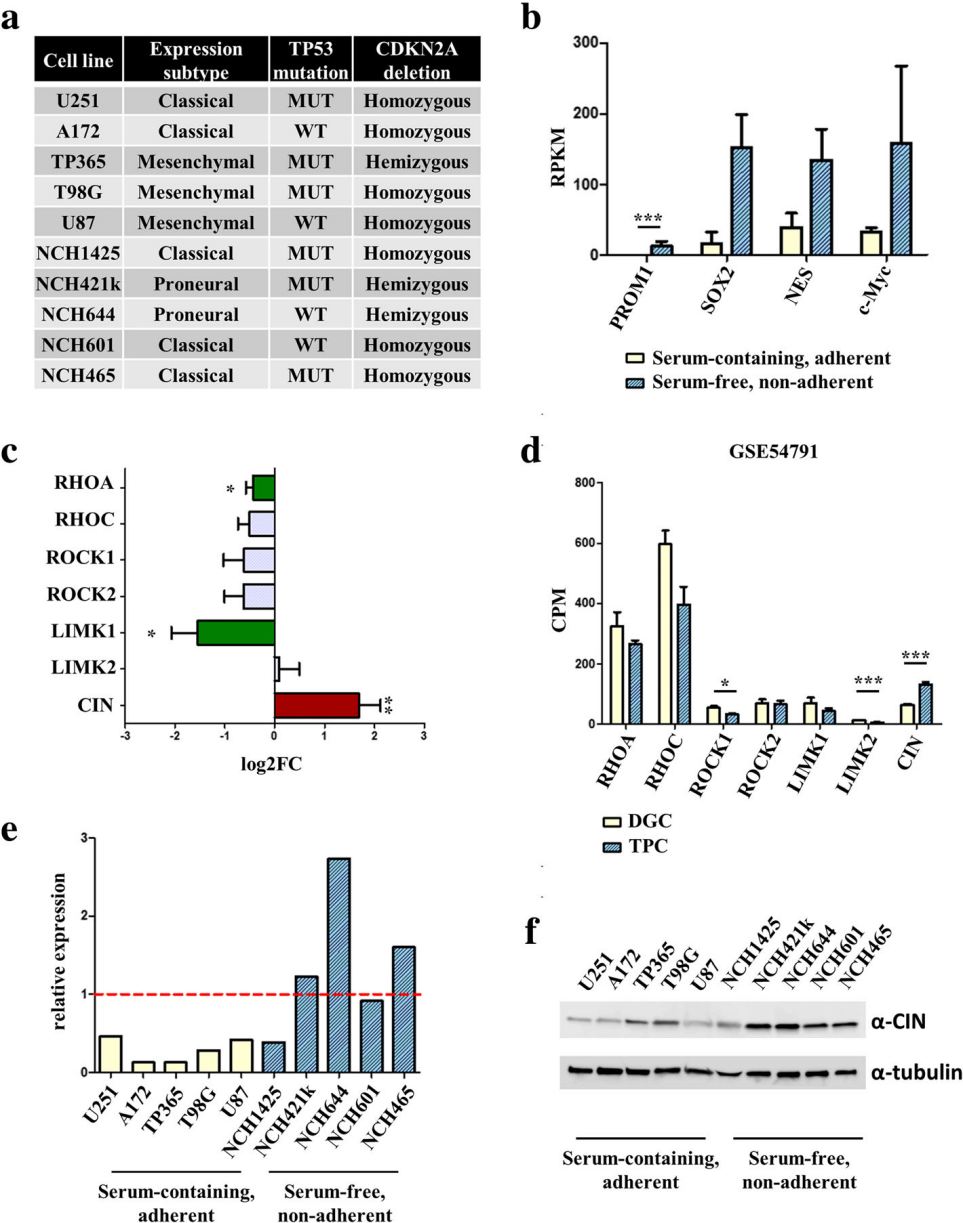
CIN shows higher expression levels in non-adherent cells cultured under serum-free conditions than in adherent lines cultured in serum-containing medium. **a** Molecular characteristics including expression subtype, presence of *TP53* mutations and *CDKN2A* deletion of all cell lines used in this study. Non-adherent cell lines cultured in serum-free medium show more often a proneural expression subtype, while mesenchymal signatures are enriched in adherent, serum-cultured cell lines. Cell lines show high frequencies of *TP53* mutations and *CDKN2A* deletions in both conditions. A more comprehensive overview of molecular characteristics including expression patterns of key glioblastoma subtype genes can be found in Additional file 1: Table S1. **b** DESeq2 analysis of stem cell markers in non-adherent cell lines cultured in serum-free medium vs. adherent, serum-cultured cell lines. The stem cell marker *PROM1* (CD133) is significantly overexpressed (DESeq2, adjusted *p*-value < 0.001). The stem cell markers SOX2, c-MYC and NES show higher expression values in cells cultured in serum-free medium. However, the differences are not significant. Shown are mean RPKM values + SD of *n* = 5 cell lines in each group. **c** DESeq2 analysis of genes regulating cofilin phosphorylation in cells cultured in serum-free medium and serum-cultured cell lines. Chronophin (CIN/PDXP) is overexpressed in glioblastoma cells cultured in serum-free medium, whereas LIMK1 is downregulated (DESeq2, adjusted *p*-value < 0.01 and *p* < 0.05, respectively). Shown is the log2 fold change of non-adherent cell lines cultured in serum-free medium versus adherent, serum-cultured cell lines + standard error. **d** Analysis of the dataset GSE54791. Similar changes as in (**c**) can be found in independently generated datasets of tumor propagating cells (TPC) and differentiated glioma cells (DGC). CIN is significantly overexpressed in glioblastoma tumor propagating cells, while LIMK2 and ROCK1 are downregulated. The *p*-values shown are Bonferroni corrected. **e** Analysis of CIN expression by real-time PCR. The expression of CIN is significantly higher (two-sided Mann-Whitney test, *p* < 0.05) in cells cultured in serum-free medium. Individual expression values of five non-adherent cell lines cultured in serum-free medium vs. five adherent, serum-cultured cell lines are shown. The expression value in normal human astrocytes was set to one (red dotted line). **f** Western blot analysis of CIN expression. The expression of CIN is high in all five cell lines cultured in serum-free medium and significantly higher in these lines compared to the adherent, serum-cultured lines (two sided t-test, *p* = 0.0005)

As a starting point, we performed an extensive molecular characterization of five non-adherent cell lines cultured in serum-free medium and five adherent cell lines cultured in serum-containing medium. For this purpose, we determined their expression subtype by next generation sequencing followed by a single sample gene set enrichment analysis. In addition, we determined *CDKN2A* deletion, *IDH1/2* mutation and the *TP53* mutational status of our cell lines (Fig. 1a and Additional File 1: Table S1). We found that *TP53* mutations were very common alterations in both serum-cultured and cell lines cultured under serum-free conditions and that all lines tested carried at least a hemizygous *CDKN2A* deletion (Fig. 1a). Interestingly, the three best characterized glioblastoma expression subtypes (proneural, classical, mesenchymal) were all present in our cell line cohort (Fig. 1a). We also confirmed that the bona fide stem cell markers *PROM1* (CD133), *NES* (nestin), *SOX2* [41] as well as *MYC* (c-Myc) were overexpressed in the cells cultured under serum-free conditions (Fig. 1b), although the difference was only significant for *PROM1* as determined by DESeq2 (adj. *p* < 0.001). When we examined differential expression patterns between serum-cultured cells and cells cultured in serum-free medium, we found that proteins regulating cofilin phosphorylation were indeed deregulated in stem-like cells, in a way favoring lower cofilin phosphorylation (Fig. 1c). This is in accordance with studies examining cofilin phosphorylation in pluripotent cells and colon cancer [9, 10, 42]. To corroborate these findings and excluding media artifacts, we reexamined the NGS-data from another study [43] that established cultures from tumor propagating glioma cells and differentiated glioma cells. Indeed, we found similar effects (Fig. 1d). Especially CIN was highly and significantly upregulated in stem-like cells (*p* < 0.001, two-sided t-test, Bonferroni-corrected). We then verified our sequencing data with real-time PCR and western blotting. There was a significant reduction in CIN mRNA as judged by real-time PCR in serum-cultured lines (two-sided Mann-Whitney test, *p* < 0.05) although levels were variable (Fig. 1e). Normal human astrocytes were used as a reference for this analysis. CIN showed a higher abundance on protein level in every cell line cultured under serum-free conditions and the difference in protein levels between the culture conditions was highly significant (Fig. 1f, *p* < 0.0005).

**Fig. 2:**
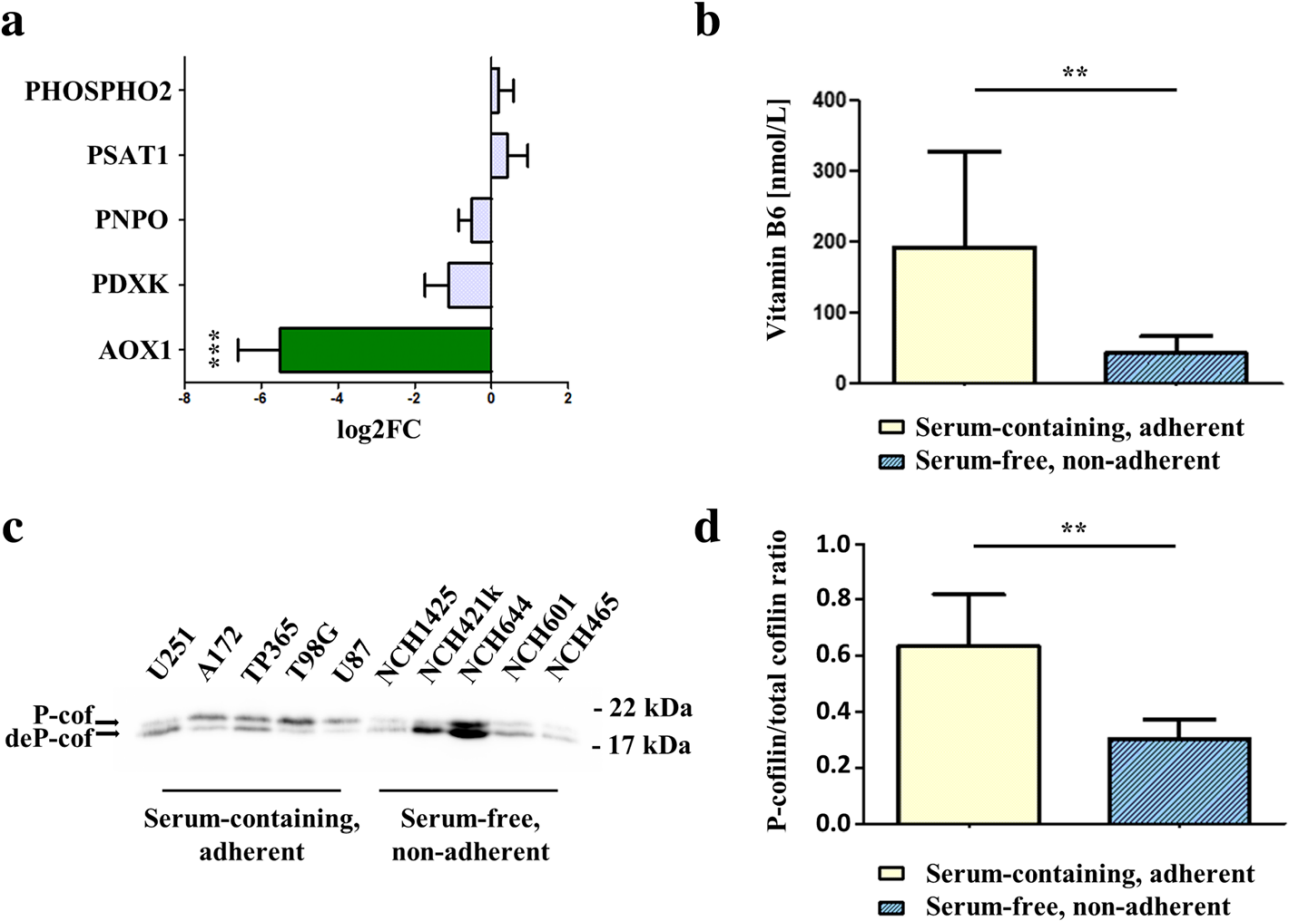
CIN substrates show higher abundance in adherent cell lines cultured in serum-containing medium than in non-adherent lines cultured in serum-free medium. **a** DESeq2 analysis of non-adherent glioblastoma cells cultured in serum-free medium vs. adherent, serum-cultured cell lines. AOX1, the enzyme that converts pyridoxal (the precursor of active pyridoxal 5′-phosphate) to 4-pyridoxate is strongly downregulated in cells cultured in serum-free medium (DESeq2, adjusted p-value< 0.001). Shown is the log2FC + standard error. **b** Quantification of active vitamin B6 levels in (*n* = 5 each) serum-cultured lines and cells cultured in serum-free medium. Active vitamin B6 levels are significantly higher (two-sided Mann-Whitney test, *p* < 0.01) in serum-cultured cell lines. **c** PhosTag western blot of glioblastoma cells grown under non-adherent, serum-free conditions and adherent, serum-cultured cell lines probed with α-cofilin antibody. **d** Quantification of *n* = 5 serum-cultured cell lines and *n* = 5 cell lines grown in serum-free medium. There is a significant increase (two-sided t-test, *p* < 0.01) in the p-cofilin/total cofilin ratio in serum-cultured cell lines

We analyzed the levels of active vitamin B6 and expression changes in the pathway regulating vitamin B6 metabolism. Active vitamin B6 levels were significantly higher in serum-cultured cell lines and the expression of AOX1 that metabolizes the precursor of pyridoxal 5′-phosphate, the active form of vitamin B6, was strongly downregulated (Fig. 2a and b). We then examined cofilin phosphorylation taking advantage of the PhosTag compound [44], which separates specifically p-Ser3-cofilin from unphosphorylated cofilin in glioma cells as established previously [8]. We found that p-cofilin levels are indeed strongly reduced in glioblastoma cell lines cultured under non-adherent, serum-free conditions (Fig. 2c and d; *p* < 0.01). Importantly, we also verified this result with a p-Ser3-cofilin specific antibody and found that results from PhosTag blotting and blotting with a p-Ser3-cofilin specific antibody were highly correlated (Additional file 2: Figure S1). We therefore hypothesized that CIN might regulate glioblastoma stem cell identity via its function in cofilin phosphorylation and/or vitamin B6 metabolism.

### Cell proliferation is not perturbed by CIN loss in vitro

We established CIN-knockdown cell lines from two stem-like cell lines representing the proneural expression subtype, NCH644 and NCH421k. CIN levels are higher in this subtype as compared to the classical and mesenchymal subtype in brain tumor samples [8] and this model system should be closest to the tumor situation.

**Fig. 3:**
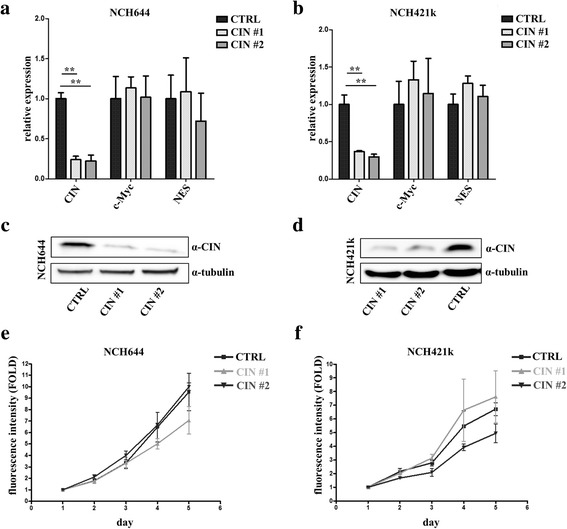
Establishment of CIN knockdown cell lines and cell proliferation analyzes. **a** and **b** Real-time PCR of CIN, c-Myc and NES in NCH644 (**a**) and NCH421k (**b**). While CIN levels are significantly reduced by two different CIN targeting shRNAs in NCH644 and NCH421k (*p* < 0.01), the mRNA levels of the glioblastoma stem cell markers c-Myc and NES are not affected. **c** and **d** Representative examples of CIN western blots in NCH644 (**c**) and NCH421k (**d**). The knockdown of CIN was efficient in both NCH644 and NCH421k. Alpha-tubulin was used as loading control. **e** and **f** Proliferation assay of shRNA knockdown cell lines in NCH644 **e** and NCH421k **f**. There are no significant differences between control and CIN shRNA cells (repeated measures two-way ANOVA followed by bonferroni corrected post-hoc tests)

There was a highly significant reduction in CIN mRNA levels, but not in the mRNA levels of the stem cell markers c-Myc or NES (two-way ANOVA, Bonferroni-corrected post-test, *p* < 0.01 in NCH644 and NCH421k for both CIN shRNA #1 and CIN shRNA #2; *p* > 0.05 for c-Myc and NES, Fig. 3a and b). However, the differentiation markers GFAP and TUBB3 were overexpressed in every knockdown cell line (Additional file 3: Figure S3). The CIN knockdown was also confirmed on protein level in both NCH644 and NCH421k (Fig. 3c and d). Proliferation was neither affected in NCH421k nor NCH644 and knockdown cell lines showed robust in vitro growth with no differences to controls (repeated measures, two-way ANOVA, Bonferroni-corrected post-hoc tests, *p* > 0.05; Fig. 3e and f).

### Active vitamin B6 levels are increased by CIN knockdown

**Fig. 4:**
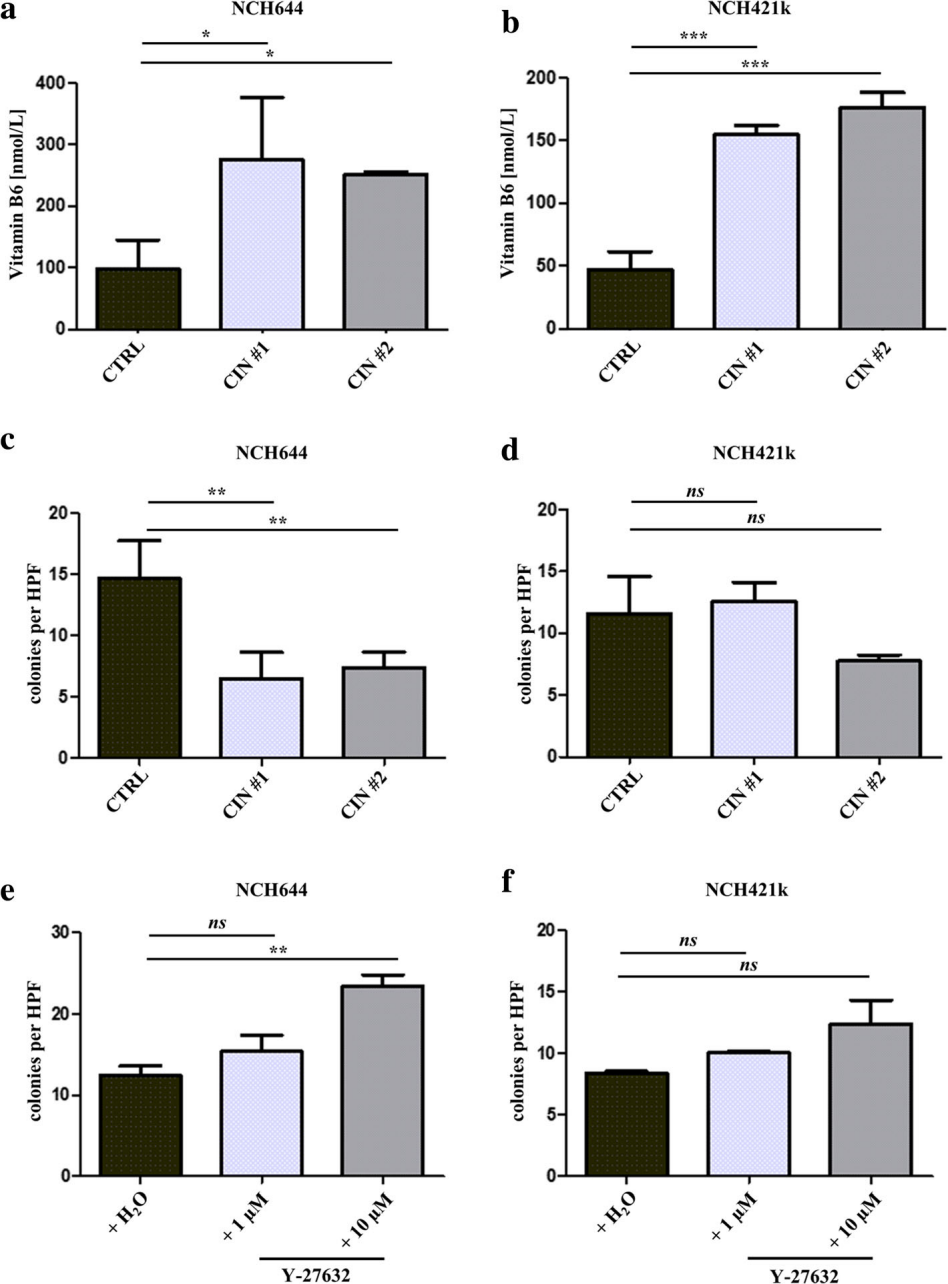
Changes in cellular phenotypes after CIN knockdown. **a** and **b** Active vitamin B6 levels are significantly increased after CIN knockdown in NCH644 **a** and NCH421k **b** (*n* = 3, one-way ANOVA followed by Dunnett’s multiple comparisons test, *p* < 0.05 and 0.001, respectively). Shown are means + SD. **c** and **d** Colony formation assay in a collagen matrix. Colony numbers are significantly reduced after CIN knockdown in NCH644 (*n* = 3, generalized linear model (poisson family with link function log), *p* < 0.01 for both CIN shRNAs) but not in NCH421k (**d**). Shown are means +SD. **e** and **f** Colony formation assay in a collagen matrix. Colony numbers are significantly increased after treatment with 10 μM Y-27632 in NCH644 **e** (*n* = 3, generalized linear model (poisson family with link function log), *p* < 0.01 for 10 μM Y-27632, *p* > 0.05 for 1 μM Y-27632) but not in NCH421k (**f**). Shown are means +SD

We then analyzed if one of the two substrates of CIN, active vitamin B6 or phosphocofilin showed altered abundance after knockdown of CIN. Indeed, active vitamin B6 levels increased significantly in NCH644 and NCH421k cells (Fig. 4a and b: one-way ANOVA followed by Dunnett’s multiple comparison’s test, *p* < 0.05 and *p* < 0.001 for both shRNAs in NCH644 and NCH421k, respectively), whereas phosphocofilin/total cofilin ratios remained unaltered (Additional file 4: Figure S2, one-way ANOVA followed by Dunnett’s multiple comparison’s test, p > 0.05). It should be noted that alterations in phosphocofilin/total cofilin ratios are often absent in serum-cultured cell lines, too [8].

### Impairment in colony formation capacity depends on genetic background

We then examined if the highly clonogenic cell lines NCH644 and NCH421k showed an alteration in colony formation ability on CIN-knockdown. Indeed, the colony numbers where significantly reduced by both CIN targeting shRNAs in NCH644 (Fig. 4c, generalized linear model, family = poisson, link-function = log, *p* < 0.01 for both shRNAs) but not in NCH421k (Fig. 4d, generalized linear model, family = poisson, link-function = log, p > 0.05 for both shRNAs). Importantly, treatment with the ROCK-inhibitor Y-27632 led to a significant increase in colony numbers (Fig. 4e, generalized linear model, family = poisson, link-function = log, *p* < 0.01 for Y-27632 at 10 μM) supporting our hypothesis that phosphoregulation of cofilin is responsible for this phenotype. In addition, in NCH421k cells, no significant phenotype was found in analogy to the CIN knockdown results.

### Next generation sequencing reveals profound changes in the cellular transcriptome after CIN downregulation

As cell proliferation itself was not perturbed by CIN knockdown, we performed poly-A RNA-Seq in NCH421k and NCH644 cells to determine if CIN might influence stem cell-related transcriptional activity. We deposited this dataset under GEO accession GSE98797. On the single gene level, quality control plots showed a high enrichment of small *p*-values after standard filtering [36], indicative of a significant effect of CIN on the cellular transcriptome and many deregulated genes (Additional files 5, 6, 7: Figure S4, Table S2 and Table S3).

**Fig. 5:**
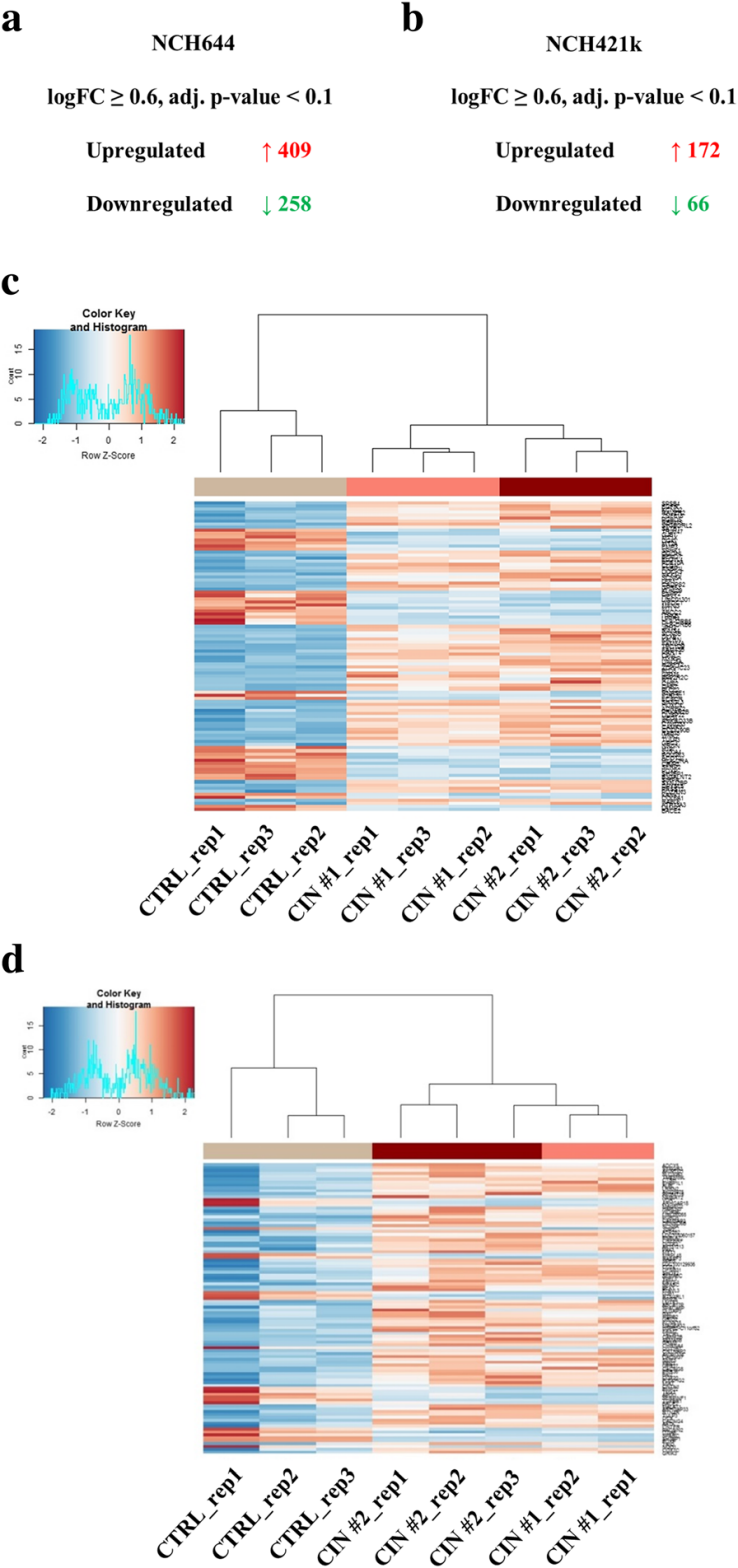
Next-generation sequencing based characterization of NCH644 and NCH421k CIN knockdown cells. **a** After CIN knockdown there are 409 upregulated and 258 downregulated genes in NCH644 CIN shRNA cells as compared to CTRL cells. **b** After CIN knockdown there are 172 upregulated and 66 downregulated genes in NCH421k CIN shRNA cells as compared to CTRL cells. **c** Heatmap of the Top100 differentially regulated genes (sorted by adjusted p-value, based on rlog normalized counts) in NCH644. Hierarchical clustering separates control cells and knockdown cells efficiently. **d** Heatmap of the Top100 differentially regulated genes (sorted by adjusted p-value, based on rlog normalized counts) in NCH421k. Hierarchical clustering separates control cells and knockdown cells efficiently

**Fig. 6:**
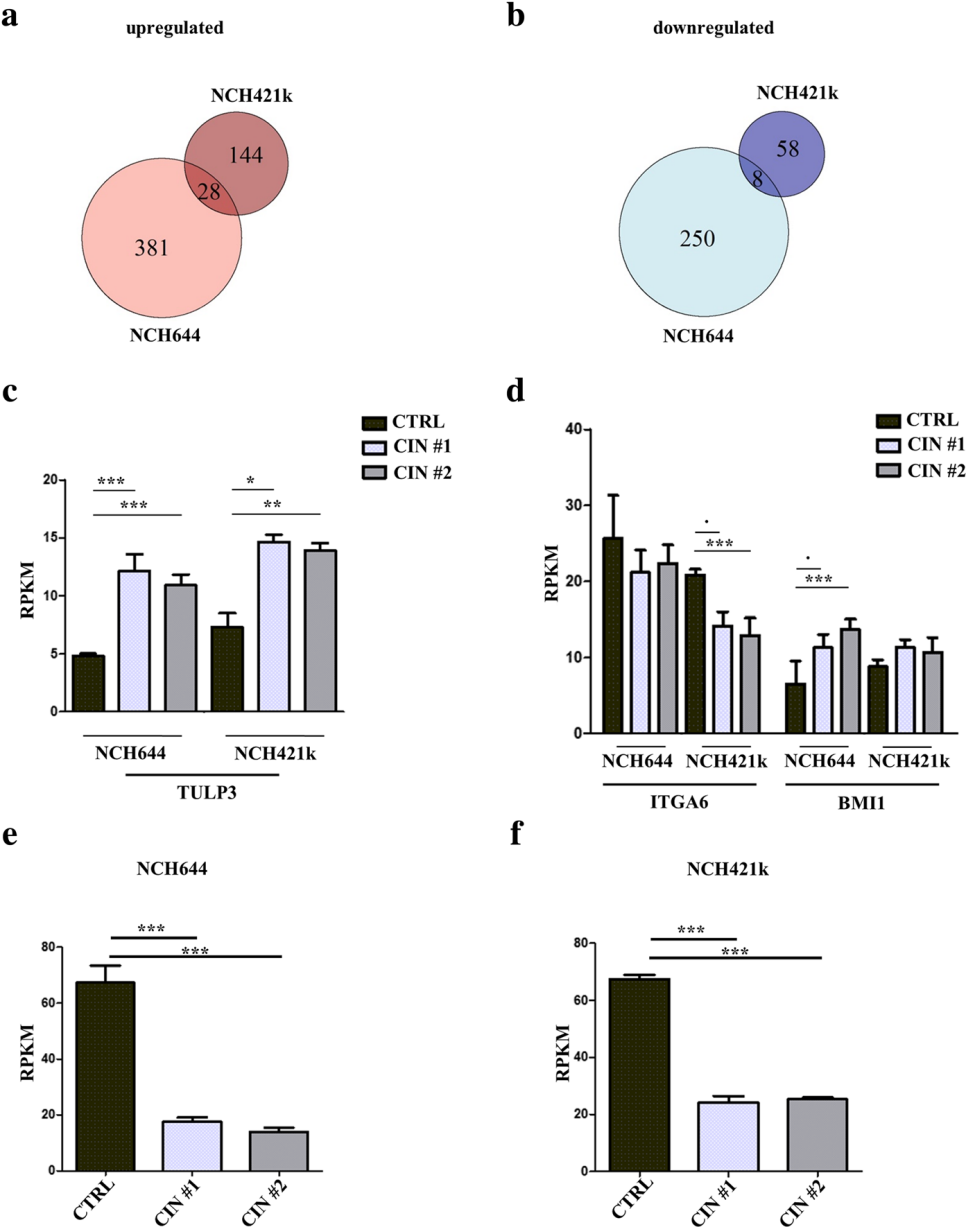
Comparison of genes of interest between NCH644 and NCH421k. **a** and **b** Venn diagrams of the NCH644 and NCH421k dataset. There is a limited overlap of 28 upregulated (**a**) and 8 downregulated (**b**) genes in both cell lines. **c** TULP3 is upregulated in both NCH644 and NCH421k after CIN knockdown. *P*-values are calculated by DESeq2, shown are RPKM means + SD. **d** ITGA6 is significantly downregulated in NCH421k after CIN knockdown, whereas BMI1 is upregulated in NCH644. P-values are calculated by DESeq2, shown are RPKM means + SD. (**e**) and (**f**) CIN is significantly downregulated in the NGS dataset in both NCH644 (**e**) and NCH421k (**f**) (one-way ANOVA followed by Dunnett’s multiple comparisons test, *p* < 0.001 for both shRNAs). Shown are means + SEM

In NCH644, there were 409 upregulated and 258 downregulated genes and in NCH421k there were 172 upregulated and 66 downregulated genes (Fig. 5a and b: logFC ≥0.6, adjusted *p* < 0.1). Hierarchical clustering based on rlog normalized expression values of the Top100 significantly deregulated genes (sorted by adjusted p-value) separated CIN shRNA and control cells efficiently in both NCH644 (Fig. 5c) as well as NCH421k (Fig. 5d). There was a limited overlap of 28 upregulated and 8 downregulated genes, when NCH644 and NCH421k were compared (Fig. 6a and b). Among the common upregulated genes was the inhibitor of hedgehog signaling TULP3 [45], which was significantly overexpressed in all CIN knockdown cell lines (Fig. 6c, adjusted *p*-values calculated by DESeq2, **· =** *p* < 0.1, * = *p* < 0.05, ** = *p* < 0.01, *** = *p* < 0.0001). The expression patterns of other glioma stem cell promoting proteins that were found in the list of deregulated genes, ITGA6 [46] and BMI1 [47], where variable. While ITGA6 was significantly downregulated in NCH421k there was only a trend in NCH644 cells. BMI1, as well as other tumor promoting genes (like EGFR and LEF1) were significantly upregulated in NCH644 in contrast to our expectations (Fig. 6d, adjusted p-values calculated by DESeq2, **· =** *p* < 0.1, * = *p* < 0.05, ** = *p* < 0.01, *** = *p* < 0.0001). CIN knockdown was highly efficient for both shRNAs in both cell lines (Fig. 6e and f, adjusted p-values calculated by DESeq2).

Global histone and DNA methylation, two possible modifications linking the transcriptomic changes and the cellular function of CIN, remained unaltered as determined by flow cytometry and pyrosequencing of LINE-1 elements (Additional file 8: Figure S5).

### ROCK and LIMK inhibitors are non-toxic for glioma cells and their influence on chemosensitivity is highly cell line-dependent

**Fig. 7:**
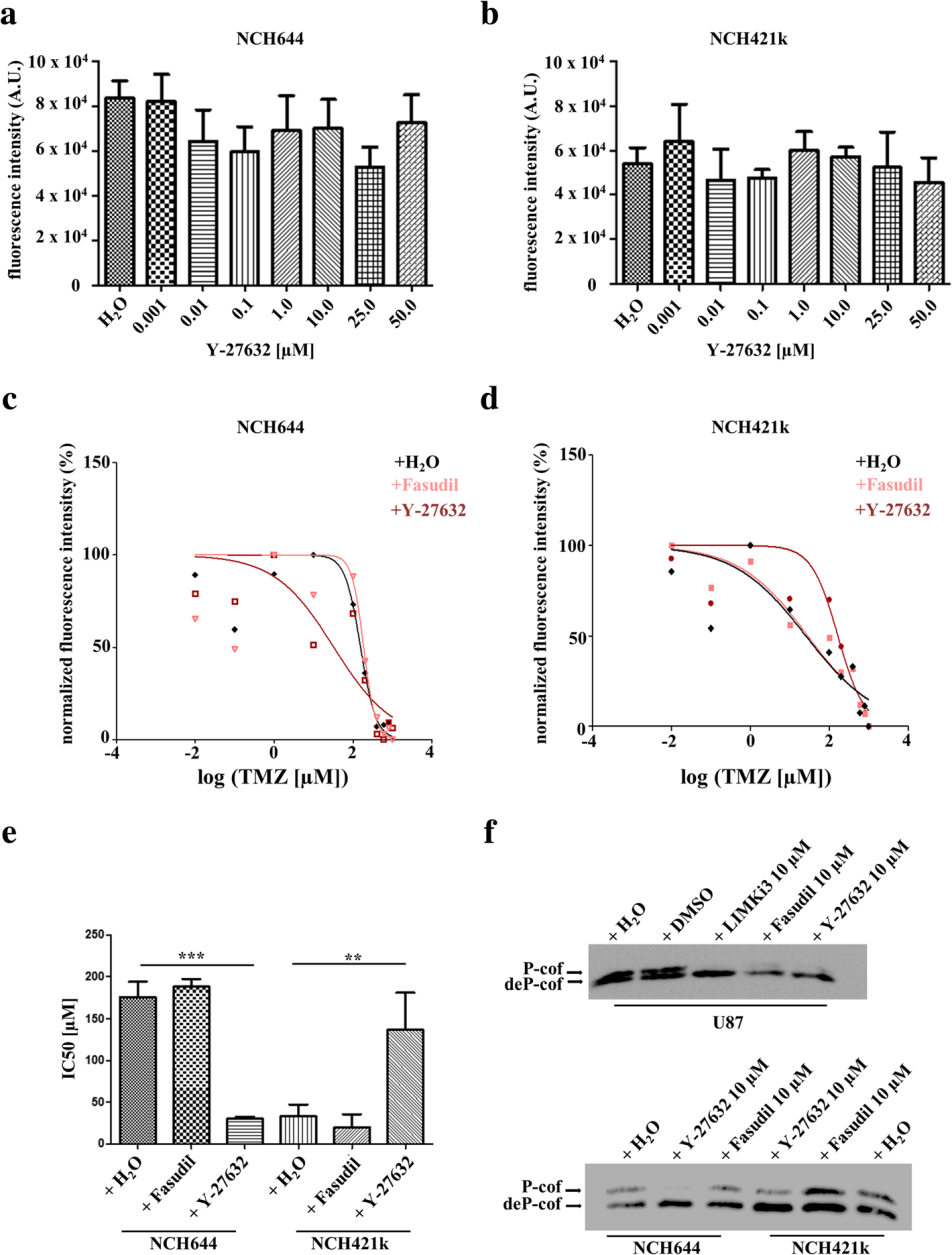
Analysis of cell viability and chemosensitivity after ROCKi treatment. **a** and **b** Measurement of cell viability with resazurin in NCH644 (**a**) and NCH421k (**b**) after Y-27632 treatment for 72 h. There is no significant decrease in cell viability after application of Y-27632 even at concentrations up to 50 μM (*n* = 3, one-way ANOVA followed by Dunnett’s multiple comparisons test, p > 0.05). Shown are means + SEM. (**c**) and (**d**) Fit of dose response curves to determine IC50 values with variable slope against the log transformed temozolomide concentration for NCH644 (**c**) and NCH421k (**d**). **e** Quantification of dose response curves as shown in (**c**) and (**d**). Temozolomide chemosensitivity changes depend on cellular background. While NCH644 cells are sensitized, NCH421k cells become more resistant by Y-27632 treatment (*n* = 3, one-way ANOVA followed by Dunnett’s multiple comparisons test, significant changes are indicated). Shown are means + SD. **f** Western blot after PhosTag gel electrophoresis probed with α-cofilin antibody. Inhibitor treatment with Y-27632 and LIMKi3 strongly reduces the levels of p-cofilin. Treatment with fasudil is less effective, especially in stem-like cells

Finally, as turnover of p-cofilin can be more important than absolute levels of the phosphorylated protein and altered absolute p-cofilin levels are not present in all cell lines cultured in serum after CIN deregulation [8], we tested if inhibitors of the upstream kinases ROCK1/2 or LIMK1/2 are able to sensitize the cell lines NCH421k and NCH644 to chemotherapeutic agents. Treatment with these inhibitors alone had an effect -if any at all- at excessively high concentrations (Fig. 7a and b, Additional file 9: Figure S6, one-way ANOVA followed by Dunnett’s multiple comparison test, significant changes indicated). However, there were opposite effects in NCH644 and NCH421k: While after inhibitor treatment of NCH644 cells there was a significant chemosensitization towards temozolomide, NCH421k cells instead showed an increase in resistance to temozolomide (Fig. 7c-e, *n* = 3, one-way ANOVA followed by Dunnett’s multiple comparisons test, significant changes are indicated). Cofilin phosphorylation in our hands was very efficiently abolished by 10 μM Y-27632 and LIMKi3 and less efficiently by fasudil in stem-like cells. U87, a classical serum cultured glioblastoma cell line with a very high phosphocofilin level was used as a positive control (Fig. 7f, Additional file 9: Figure S6). In contrast, there was no change in chemosensitivity after CIN knockdown (Additional file 10: Figure S7, one-way ANOVA followed by Dunnett’s multiple comparison test, significant changes indicated).

## Discussion

It was previously shown that cell lines cultured in serum in which CIN is targeted by a shRNA grow much slower in vivo than their CIN expressing counterparts [8] suggesting a loss of stemness in CIN deficient cells. Indeed, we found that CIN is strongly overexpressed in glioblastoma cells cultured under serum-free, non-adherent conditions which are thought to be permissive for stem-like cells [12], in contrast to cell lines cultured in serum-containing medium. It should be noted that, as we did not compare genetically matched lines in different media, effects of the genetic background on the phenotypes cannot be entirely excluded. In addition, LIMK1 that catalyzes cofilin phosphorylation was downregulated in cells grown under serum-free conditions in line with reports that provide evidence for a stemness-promoting role of unphosphorylated cofilin [9, 10]. The two canonical cellular targets of CIN, pyridoxal 5′-phosphate [14], the active form of vitamin B6, and p-Ser3-cofilin [11] are strongly reduced in glioblastoma cells cultured in serum-free medium. When modelling CIN loss in vitro, we found an increase in active vitamin B6, a loss of colony formation ability in one out of two cell lines but no change in phosphocofilin levels. The latter might be explained by the fact that our cells grow as neurospheres and that changes in phosphocofilin induced by CIN are only induced in dependence on cell adhesion. In support of our hypothesis, treatment with Y-27632, a ROCK-inhibitor, induced the opposite phenotype with respect to colony formation. In addition, alterations in the phosphocofilin/total cofilin ratio are often absent in serum-cultured cell lines, too [8]. It should be noted that CIN knockdown specifically promotes colony formation in serum-cultured cells despite the loss of in vivo growth capacity [8]. This finding underscores that cells cultured under serum-free conditions and overexpressing the stemness marker *PROM1* -as utilized in our present investigation- better mimic the in vivo situation.

The observed changes in active vitamin B6 levels potentially lead to global alterations in metabolism, as vitamin B6 is a cofactor in numerous enzymatic reactions [15]. A reduction in dietary vitamin B6 is used to reduce global DNA-methylation in mice. Vitamin B6 is a cofactor in SAM biosynthesis which is the main methyl donor for DNA- and histone- methylation [48]. However, in our analyzes we did not observe any changes in DNA- or histone-methylation, suggesting that vitamin B6 is not a limiting factor for SAM biosynthesis in glioblastoma stem-like cells. There are several clues suggesting an association between CIN and chemotherapy resistance in glioblastoma cells: As such, stemness and chemoresistance are supposed to be linked in glioblastoma [49], CIN regulates the actin cytoskeleton in response to genotoxic stress [50] and vitamin B6 metabolism is an important factor for chemoresistance in lung cancer [21] that regulates cisplatin accumulation in vitro [51]. Despite all those hints, the resistance to temozolomide remained unaltered after CIN knockdown in both cell lines examined. The lack of epigenetic vitamin B6 effects uncovered in this study may at least provide one possible explanation for this phenomenon.

There were significant changes on the single gene level after CIN knockdown, which are, however, difficult to interpret with respect to stemness. While some stemness promoting genes like EGFR, BMI1 and even LEF1 were upregulated in NCH644, others like HES1 and HEY1 were downregulated, suggesting a complex relationship between CIN effects on the regulation of stemness pathways and/or compensatory reactions within the cell lines. One of the identified genes, TULP3, an inhibitor of hedgehog signaling [52] caught our attention, as CIN had been reported to be transcriptionally altered in response to inhibition of hedgehog signaling in colon cancer cells [53]. Also, hedgehog pathway is important for glioblastoma stem cell self-renewal [54]. We also noted that besides TULP3, many other proteins that regulate neuronal functions in health and disease, e.g. *CAMK2B* [55], *TTBK1* [56] and *LRRN2* [57], were deregulated in both cell lines suggesting a function of CIN in neuronal development and (dys)function.

ROCK-inhibitors have been proposed to facilitate glioblastoma stem-like cell [23] and pluripotent stem cell expansion [58]. Others have shown strong pro-apoptotic action of LIMK-inhibitors in breast cancer cells [20] and of ROCK-inhibitors on serum-cultured glioma cells [59]. Also, ROCK inhibitors have been suggested as effective against leukemia [60]. In assessing the potential use of these molecules in glioblastoma treatment we tested their effectiveness in influencing cell viability of glioblastoma cells with or without the addition of alkylating chemotherapeutics (temozolomide). ROCK- and LIMK-inhibitors alone did not influence cell viability except at excessive concentrations ≥25 μM. However, they were well able to boost colony formation. In addition, although we were able to show effective removal of p-Ser3-cofilin in all cell lines, the responses towards chemotherapeutic agents in presence of the inhibitors were contrary between cell lines. Thus, use of these compounds needs further clarification and a better understanding of the mechanism by which cofilin regulates apoptosis.

## Conclusion

Cofilin phosphorylation and Chronophin expression differ between adherent cell lines cultured in serum-containing medium and non-adherent glioblastoma cell lines cultured in serum-free medium. Chronophin knockdown in the latter setting induces phenotypic changes, e.g. in colony formation and transcription, but these are highly dependent on the cellular background. The same is true for phenotypes observed after treatment with inhibitors for kinases regulating cofilin phosphorylation, ROCKs and LIMKs. Targeting the cofilin phosphorylation pathway might therefore not be a straightforward therapeutic option in glioblastoma.

## Additional files

Additional file 1: **Table S1.** Comprehensive molecular characterization of glioblastoma cell lines. Comprehensive molecular characterization of all cell lines used in this study based on RNA-Seq. This table contains the name of the cell lines, mutation and expression analysis for TP53, analysis of genomic deletion (based on real-time PCR) and expression for CDKN2A as well as expression data for several glioblastoma subtype-specific genes. (XLSX 12 kb)

Additional file 2: **Figure S1.** P-Ser3-cofilin and cofilin western blotting. (A) Western blots of lysates from non-adherent cells cultured in serum-free medium and serum-cultured cell lines probed with α-Ser3-phosphocofilin, α-cofilin and α-tubulin antibody. (B) Quantification of *n* = 5 cell lines in each group as shown in (A). There is a significant increase (two-tailed t-test, *p* < 0.05) in the p-Ser3-cofilin signal relative to total cofilin in serum-cultured cell lines. (C) Correlation analysis of standard western blotting and PhosTag western blot. Quantifications from both methods are highly correlated. (TIF 436 kb)

Additional file 3: **Figure S2.** Differentiation markers are induced upon CIN depletion. (A) and (B) Real-time PCR of the differentiation markers GFAP and TUBB3 in NCH644 (A) and NCH421k (B). There are significant differences between the groups for GFAP and TUBB3 in NCH644 as well as for GFAP in NCH421k (one-way anova, *p* < 0.05 for all comparisons mentioned, *n* = 2). (TIF 294 kb)

Additional file 4: **Figure S3.** Analysis of phosphocofilin levels in CIN knockdown cell lines. (A) Representative example of a western blot after PhosTag gel electrophoresis probed with α-cofilin antibody. (B) and (C) Quantification of western blots as shown in (A) for NCH644 (B) and NCH421k (C). There is no significant difference in the P-cofilin/total cofilin ratio between CTRL or CIN shRNA cells (*n* = 3, one-way ANOVA followed by Dunnett’s multiple comparisons test, *p* > 0.05). Shown are means + SD. (TIF 328 kb)

Additional file 5: **Figure S4.** Quality control plots for next generation sequencing data. (A) and (C) There is a high enrichment of small *p*-values after standard filtering in both NCH644 (A) and NCH421k (C) as calculated by DESeq2. (B) and (D) MA-plots indicate the presence of many deregulated genes (red) in both NCH644 (B) and NCH421k (D). (TIF 1008 kb)

Additional file 6: **Table S2.** Genes deregulated in NCH644 after CIN knockdown (NGS results). Table of the results from the NGS analysis for NCH644 (comparison CTRL vs. CIN shRNA #1 and #2) including statistics as calculated by DESeq2. (XLSX 5446 kb)

Additional file 7: **Table S3.** Genes deregulated in NCH421k after CIN knockdown (NGS results). Table of the results from the NGS analysis for NCH421k (comparison CTRL vs. CIN shRNA #1 and #2) including statistics as calculated by DESeq2. (XLSX 5355 kb)

Additional file 8: **Figure S5.** Flow cytometry-based analysis of histone methylation levels and pyrosequencing-based analysis of global DNA methylation. (A) Exemplary sequencing run of a LINE-1 sequence from the Pyromark instrument. (B) Quantification of global DNA methylation profiles by LINE-1 element pyrosequencing in NCH644 and NCH421k (*n* = 3, one-way ANOVA followed by Dunnett’s multiple comparisons test, *p* > 0.05). Shown are means + SD. (C) Quantification of flow cytometry experiments of NCH644 cells stained with H3K27me3 or control IgG. Levels of histone H3 trimethylated at lysine 27 remain unchanged after CIN knockdown (*n* = 3, one-way ANOVA followed by Dunnett’s multiple comparisons test, p > 0.05). Shown are geometric means + SD. (TIF 893 kb)

Additional file 9: **Figure S6.** Analysis of cell viability after LIMKi and fasudil treatment. (A) and (B) Measurement of cell viability with resazurin in NCH644 (A) and NCH421k (B) after LIMKi3 inhibitor treatment for 72 h. There is no significant decrease in cell viability after LIMKi3 application even at concentrations up to 50 μM (*n* = 3, one-way ANOVA followed by Dunnett’s multiple comparisons test, p > 0.05). Shown are means + SEM. (C) and (D) Measurement of cell viability with resazurin in NCH644 (C) and NCH421k (D) after fasudil treatment. A significant decrease in cell viability occurred only in NCH421k at the highest concentrations of 25 and 50 μM (*n* = 3, one-way ANOVA followed by Dunnett’s multiple comparisons test, *p* < 0.01 and 0.001, respectively). Shown are means + SEM. (E) Western blot after PhosTag gel electrophoresis probed with α-cofilin antibody. Inhibitor treatment with LIMKi3 strongly reduces the levels of p-cofilin. (TIF 1192 kb)

Additional file 10: **Figure S7.** Analysis of chemosensitivity in CIN knockdown cell lines. Temozolomide chemosensitivity remains unaltered after CIN knockdown. In both NCH644 and NCH421k there is no significant difference in IC50 values after CIN knockdown (*n* = 3, one-way ANOVA followed by Dunnett’s multiple comparisons test, *p* > 0.05). Shown are means + SD. (TIF 256 kb)

## Abbreviations

Adj: Adjusted
ANOVA: Analysis of variance
bFGF2: Basic fibroblast growth factor 2
CIN: Chronophin
DGC: Differentiated glioma cells
EGF: Epidermal growth factor
FDR: False discovery rate
HPLC: High-performance liquid chromatography;
LIMK: LIM domain kinase
LINE: Long interspersed nuclear element
log2FC: Log2 fold change
MA-plot: Plot of log2FCs over the mean of normalized counts
NES: Normalized enrichment score
NP: Nominal *p*-value
ROCK: Rho associated coiled-coil containing protein kinase
RPKM: Reads per kilobase of exon model per million mapped reads
shRNA: Short hairpin RNA
TPC: Tumor propagating cells

## References

[1] D'Abaco GM, Kaye AH (2007). Integrins: molecular determinants of glioma invasion. J Clin Neurosci.

[2] Condeelis J, Singer RH, Segall JE (2005). The great escape: when cancer cells hijack the genes for chemotaxis and motility. Annu Rev Cell Dev Biol.

[3] Nagai S, Moreno O, Smith CA, Ivanchuk S, Romagnuolo R, Golbourn B, Weeks A, Seol HJ, Rutka JT (2011). Role of the cofilin activity cycle in astrocytoma migration and invasion. Genes Cancer.

[4] Bravo-Cordero JJ, Magalhaes MA, Eddy RJ, Hodgson L, Condeelis J (2013). Functions of cofilin in cell locomotion and invasion. Nat Rev Mol Cell Biol.

[5] Bugyi B, Carlier MF (2010). Control of actin filament treadmilling in cell motility. Annu Rev Biophys.

[6] Mizuno K (2013). Signaling mechanisms and functional roles of cofilin phosphorylation and dephosphorylation. Cell Signal.

[7] Manetti F (2012). LIM kinases are attractive targets with many macromolecular partners and only a few small molecule regulators. Med Res Rev.

[8] Schulze M, Fedorchenko O, Zink TG, Knobbe-Thomsen CB, Kraus S, Schwinn S, Beilhack A, Reifenberger G, Monoranu CM, Siren AL (2016). Chronophin is a glial tumor modifier involved in the regulation of glioblastoma growth and invasiveness. Oncogene.

[9] Sakurai K, Talukdar I, Patil VS, Dang J, Li Z, Chang KY, Lu CC, Delorme-Walker V, Dermardirossian C, Anderson K (2014). Kinome-wide functional analysis highlights the role of cytoskeletal remodeling in somatic cell reprogramming. Cell Stem Cell.

[10] Lourenco FC, Munro J, Brown J, Cordero J, Stefanatos R, Strathdee K, Orange C, Feller SM, Sansom OJ, Vidal M (2014). Reduced LIMK2 expression in colorectal cancer reflects its role in limiting stem cell proliferation. Gut.

[11] Gohla A, Birkenfeld J, Bokoch GM (2005). Chronophin, a novel HAD-type serine protein phosphatase, regulates cofilin-dependent actin dynamics. Nat Cell Biol.

[12] Campos B, Gal Z, Baader A, Schneider T, Sliwinski C, Gassel K, Bageritz J, Grabe N, von Deimling A, Beckhove P (2014). Aberrant self-renewal and quiescence contribute to the aggressiveness of glioblastoma. J Pathol.

[13] Lee J, Kotliarova S, Kotliarov Y, Li A, Su Q, Donin NM, Pastorino S, Purow BW, Christopher N, Zhang W (2006). Tumor stem cells derived from glioblastomas cultured in bFGF and EGF more closely mirror the phenotype and genotype of primary tumors than do serum-cultured cell lines. Cancer Cell.

[14] Fonda ML (1992). Purification and characterization of vitamin B6-phosphate phosphatase from human erythrocytes. J Biol Chem.

[15] Wu XY, Lu L (2012). Vitamin B6 deficiency, genome instability and cancer. Asian Pac J Cancer Prev.

[16] Kinnaird A, Zhao S, Wellen KE, Michelakis ED (2016). Metabolic control of epigenetics in cancer. Nat Rev Cancer.

[17] Ceccarelli M, Barthel FP, Malta TM, Sabedot TS, Salama SR, Murray BA, Morozova O, Newton Y, Radenbaugh A, Pagnotta SM (2016). Molecular profiling reveals biologically discrete subsets and pathways of progression in diffuse glioma. Cell.

[18] Sturm D, Bender S, Jones DT, Lichter P, Grill J, Becher O, Hawkins C, Majewski J, Jones C, Costello JF (2014). Paediatric and adult glioblastoma: multiform (epi)genomic culprits emerge. Nat Rev Cancer.

[19] Richichi C, Osti D, Del Bene M, Fornasari L, Patane M, Pollo B, DiMeco F, Pelicci G (2016). Tumor-initiating cell frequency is relevant for glioblastoma aggressiveness. Oncotarget.

[20] Croft DR, Crighton D, Samuel MS, Lourenco FC, Munro J, Wood J, Bensaad K, Vousden KH, Sansom OJ, Ryan KM (2011). p53-mediated transcriptional regulation and activation of the actin cytoskeleton regulatory RhoC to LIMK2 signaling pathway promotes cell survival. Cell Res.

[21] Galluzzi L, Vitale I, Senovilla L, Olaussen KA, Pinna G, Eisenberg T, Goubar A, Martins I, Michels J, Kratassiouk G (2012). Prognostic impact of vitamin B6 metabolism in lung cancer. Cell Rep.

[22] Petrilli A, Copik A, Posadas M, Chang LS, Welling DB, Giovannini M, Fernandez-Valle C (2014). LIM domain kinases as potential therapeutic targets for neurofibromatosis type 2. Oncogene.

[23] Tilson SG, Haley EM, Triantafillu UL, Dozier DA, Langford CP, Gillespie GY, Kim Y (2015). ROCK inhibition facilitates in vitro expansion of glioblastoma stem-like cells. PLoS One.

[24] Ohgushi M, Matsumura M, Eiraku M, Murakami K, Aramaki T, Nishiyama A, Muguruma K, Nakano T, Suga H, Ueno M (2010). Molecular pathway and cell state responsible for dissociation-induced apoptosis in human pluripotent stem cells. Cell Stem Cell.

[25] Livak KJ, Schmittgen TD (2001). Analysis of relative gene expression data using real-time quantitative PCR and the 2(−Delta Delta C(T)) method. Methods.

[26] Lin L, Goke J, Cukuroglu E, Dranias MR, VanDongen AM, Stanton LW (2016). Molecular features underlying neurodegeneration identified through in vitro modeling of genetically diverse Parkinson's disease patients. Cell Rep.

[27] Labuhn M, Jones G, Speel EJ, Maier D, Zweifel C, Gratzl O, Van Meir EG, Hegi ME, Merlo A (2001). Quantitative real-time PCR does not show selective targeting of p14(ARF) but concomitant inactivation of both p16(INK4A) and p14(ARF) in 105 human primary gliomas. Oncogene.

[28] Berggren P, Kumar R, Sakano S, Hemminki L, Wada T, Steineck G, Adolfsson J, Larsson P, Norming U, Wijkstrom H (2003). Detecting homozygous deletions in the CDKN2A(p16(INK4a))/ARF(p14(ARF)) gene in urinary bladder cancer using real-time quantitative PCR. Clin Cancer Res.

[29] Rooney JP, Ryde IT, Sanders LH, Howlett EH, Colton MD, Germ KE, Mayer GD, Greenamyre JT, Meyer JN (2015). PCR based determination of mitochondrial DNA copy number in multiple species. Methods Mol Biol.

[30] Sellick CA, Hansen R, Stephens GM, Goodacre R, Dickson AJ (2011). Metabolite extraction from suspension-cultured mammalian cells for global metabolite profiling. Nat Protoc.

[31] Fonda ML, Trauss C, Guempel UM (1991). The binding of pyridoxal 5′-phosphate to human serum albumin. Arch Biochem Biophys.

[32] Rybak ME, Jain RB, Pfeiffer CM (2005). Clinical vitamin B6 analysis: an interlaboratory comparison of pyridoxal 5′-phosphate measurements in serum. Clin Chem.

[33] Yang AS, Estecio MR, Doshi K, Kondo Y, Tajara EH, Issa JP (2004). A simple method for estimating global DNA methylation using bisulfite PCR of repetitive DNA elements. Nucleic Acids Res.

[34] Hoja S, Schulze M, Rehli M, Proescholdt M, Herold-Mende C, Hau P, Riemenschneider MJ (2016). Molecular dissection of the valproic acid effects on glioma cells. Oncotarget.

[35] Huber W, Carey VJ, Gentleman R, Anders S, Carlson M, Carvalho BS, Bravo HC, Davis S, Gatto L, Girke T (2015). Orchestrating high-throughput genomic analysis with Bioconductor. Nat Methods.

[36] Love MI, Huber W, Anders S (2014). Moderated estimation of fold change and dispersion for RNA-seq data with DESeq2. Genome Biol.

[37] Subramanian A, Tamayo P, Mootha VK, Mukherjee S, Ebert BL, Gillette MA, Paulovich A, Pomeroy SL, Golub TR, Lander ES (2005). Gene set enrichment analysis: a knowledge-based approach for interpreting genome-wide expression profiles. Proc Natl Acad Sci U S A.

[38] Reich M, Liefeld T, Gould J, Lerner J, Tamayo P, Mesirov JP (2006). GenePattern 2.0. Nat Genet.

[39] Mortazavi A, Williams BA, McCue K, Schaeffer L, Wold B (2008). Mapping and quantifying mammalian transcriptomes by RNA-Seq. Nat Methods.

[40] Verhaak RG, Hoadley KA, Purdom E, Wang V, Qi Y, Wilkerson MD, Miller CR, Ding L, Golub T, Mesirov JP (2010). Integrated genomic analysis identifies clinically relevant subtypes of glioblastoma characterized by abnormalities in PDGFRA, IDH1, EGFR, and NF1. Cancer Cell.

[41] Gunther HS, Schmidt NO, Phillips HS, Kemming D, Kharbanda S, Soriano R, Modrusan Z, Meissner H, Westphal M, Lamszus K (2008). Glioblastoma-derived stem cell-enriched cultures form distinct subgroups according to molecular and phenotypic criteria. Oncogene.

[42] Qin H, Diaz A, Blouin L, Lebbink RJ, Patena W, Tanbun P, LeProust EM, McManus MT, Song JS, Ramalho-Santos M (2014). Systematic identification of barriers to human iPSC generation. Cell.

[43] Suva ML, Rheinbay E, Gillespie SM, Patel AP, Wakimoto H, Rabkin SD, Riggi N, Chi AS, Cahill DP, Nahed BV (2014). Reconstructing and reprogramming the tumor-propagating potential of glioblastoma stem-like cells. Cell.

[44] Kinoshita E, Kinoshita-Kikuta E, Koike T (2009). Separation and detection of large phosphoproteins using Phos-tag SDS-PAGE. Nat Protoc.

[45] Cameron DA, Pennimpede T, Petkovich M (2009). Tulp3 is a critical repressor of mouse hedgehog signaling. Dev Dyn.

[46] Lathia JD, Gallagher J, Heddleston JM, Wang J, Eyler CE, Macswords J, Wu Q, Vasanji A, McLendon RE, Hjelmeland AB (2010). Integrin alpha 6 regulates glioblastoma stem cells. Cell Stem Cell.

[47] Gargiulo G, Cesaroni M, Serresi M, de Vries N, Hulsman D, Bruggeman SW, Lancini C, van Lohuizen M (2013). In vivo RNAi screen for BMI1 targets identifies TGF-beta/BMP-ER stress pathways as key regulators of neural- and malignant glioma-stem cell homeostasis. Cancer Cell.

[48] Fuso A, Nicolia V, Cavallaro RA, Ricceri L, D'Anselmi F, Coluccia P, Calamandrei G, Scarpa S (2008). B-vitamin deprivation induces hyperhomocysteinemia and brain S-adenosylhomocysteine, depletes brain S-adenosylmethionine, and enhances PS1 and BACE expression and amyloid-beta deposition in mice. Mol Cell Neurosci.

[49] Chen J, Li Y, Yu TS, McKay RM, Burns DK, Kernie SG, Parada LF (2012). A restricted cell population propagates glioblastoma growth after chemotherapy. Nature.

[50] Lee SJ, Park JW, Kang BS, Lee DS, Lee HS, Choi S, Kwon OS. The Chronophin activation is necessary in doxorubicin-induced actin cytoskeleton alteration. BMB Rep. 2017;50(6):335–40. 10.5483/BMBRep.2017.50.6.061 PMC5498145 28502289

[51] Galluzzi L, Marsili S, Vitale I, Senovilla L, Michels J, Garcia P, Vacchelli E, Chatelut E, Castedo M, Kroemer G (2013). Vitamin B6 metabolism influences the intracellular accumulation of cisplatin. Cell Cycle.

[52] Norman RX, Ko HW, Huang V, Eun CM, Abler LL, Zhang Z, Sun X, Eggenschwiler JT (2009). Tubby-like protein 3 (TULP3) regulates patterning in the mouse embryo through inhibition of hedgehog signaling. Hum Mol Genet.

[53] Shi T, Mazumdar T, Devecchio J, Duan ZH, Agyeman A, Aziz M, Houghton JA. cDNA microarray gene expression profiling of hedgehog signaling pathway inhibition in human colon cancer cells. PLoS One. 2010;5(10). 10.1371/journal.pone.0013054 PMC2948497 20957031

[54] Clement V, Sanchez P, de Tribolet N, Radovanovic I, Ruiz i, Altaba A (2007). HEDGEHOG-GLI1 signaling regulates human glioma growth, cancer stem cell self-renewal, and tumorigenicity. Curr Biol.

[55] Wayman GA, Lee YS, Tokumitsu H, Silva AJ, Soderling TR (2008). Calmodulin-kinases: modulators of neuronal development and plasticity. Neuron.

[56] Lund H, Cowburn RF, Gustafsson E, Stromberg K, Svensson A, Dahllund L, Malinowsky D, Sunnemark D (2013). Tau-tubulin kinase 1 expression, phosphorylation and co-localization with phospho-Ser422 tau in the Alzheimer's disease brain. Brain Pathol.

[57] Almeida A, Zhu XX, Vogt N, Tyagi R, Muleris M, Dutrillaux AM, Dutrillaux B, Ross D, Malfoy B, Hanash S (1998). GAC1, a new member of the leucine-rich repeat superfamily on chromosome band 1q32.1, is amplified and overexpressed in malignant gliomas. Oncogene.

[58] Street CA, Bryan BA (2011). Rho kinase proteins--pleiotropic modulators of cell survival and apoptosis. Anticancer Res.

[59] Deng L, Li G, Li R, Liu Q, He Q, Zhang J (2010). Rho-kinase inhibitor, fasudil, suppresses glioblastoma cell line progression in vitro and in vivo. Cancer Biol Ther.

[60] Oku Y, Tareyanagi C, Takaya S, Osaka S, Ujiie H, Yoshida K, Nishiya N, Uehara Y (2014). Multimodal effects of small molecule ROCK and LIMK inhibitors on mitosis, and their implication as anti-leukemia agents. PLoS One.

