# Supplementary figures and images for: Chronophin regulates active vitamin B6 levels and transcriptomic features of glioblastoma cell lines cultured under non-adherent, serum-free conditions
Source: BMC Cancer. 2018 May 3;18:524. doi: 10.1186/s12885-018-4440-4 (PMC5934884; doi:10.1186/s12885-018-4440-4)

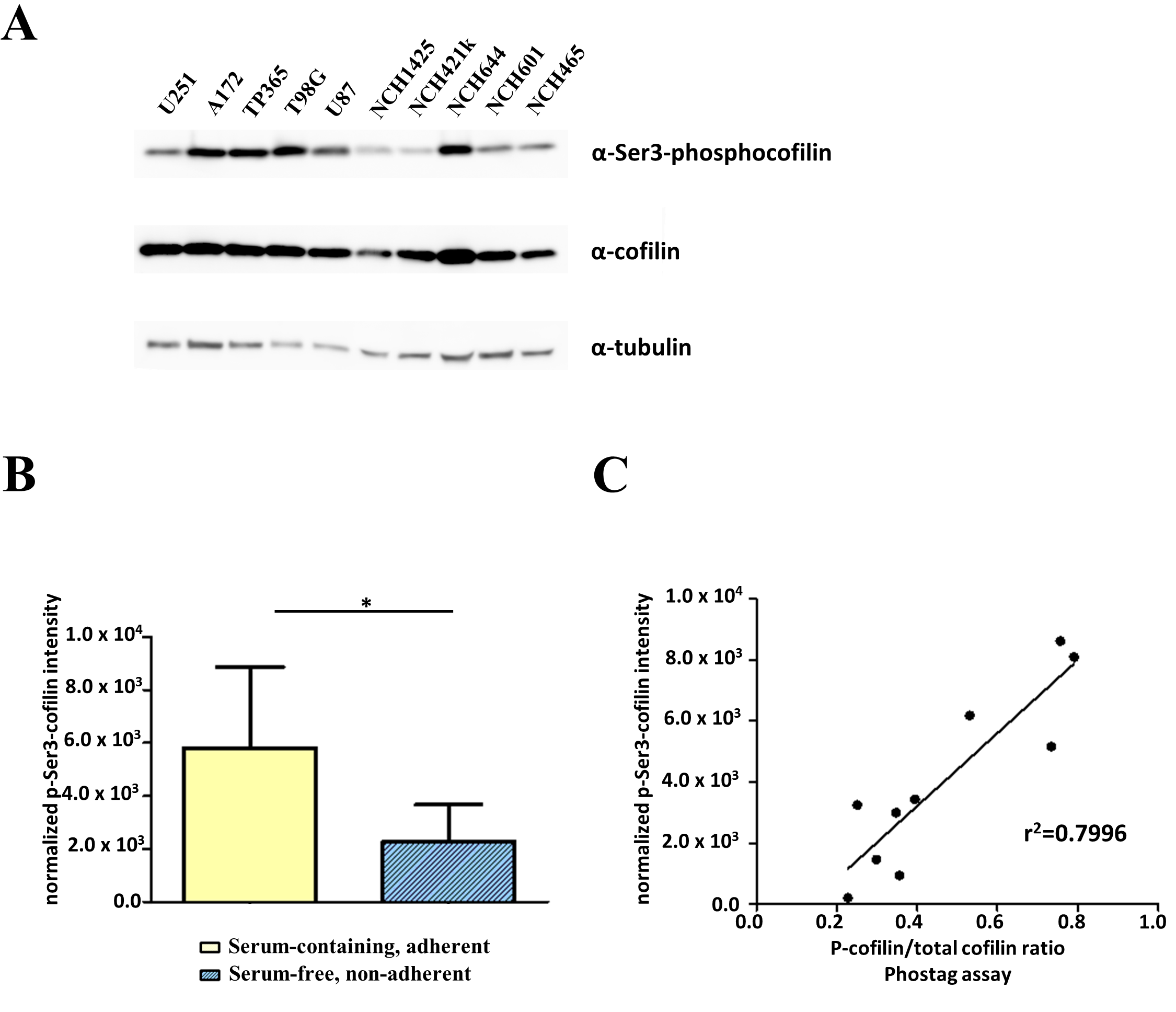

Supplement: Supplementary file 2 — Figure S1. P-Ser3-cofilin and cofilin western blotting. (A) Western blots of lysates from non-adherent cells cultured in serum-free medium and serum-cultured cell lines probed with α-Ser3-phosphocofilin, α-cofilin and α-tubulin antibody. (B) Quantification of n = 5 cell lines in each group as shown in (A). There is a significant increase (two-tailed t-test, p < 0.05) in the p-Ser3-cofilin signal relative to total cofilin in serum-cultured cell lines. (C) Correlation analysis of standard western blotting and PhosTag western blot. Quantifications from both methods are highly correlated. (TIF 436 kb) [file 12885_2018_4440_MOESM2_ESM.tif]

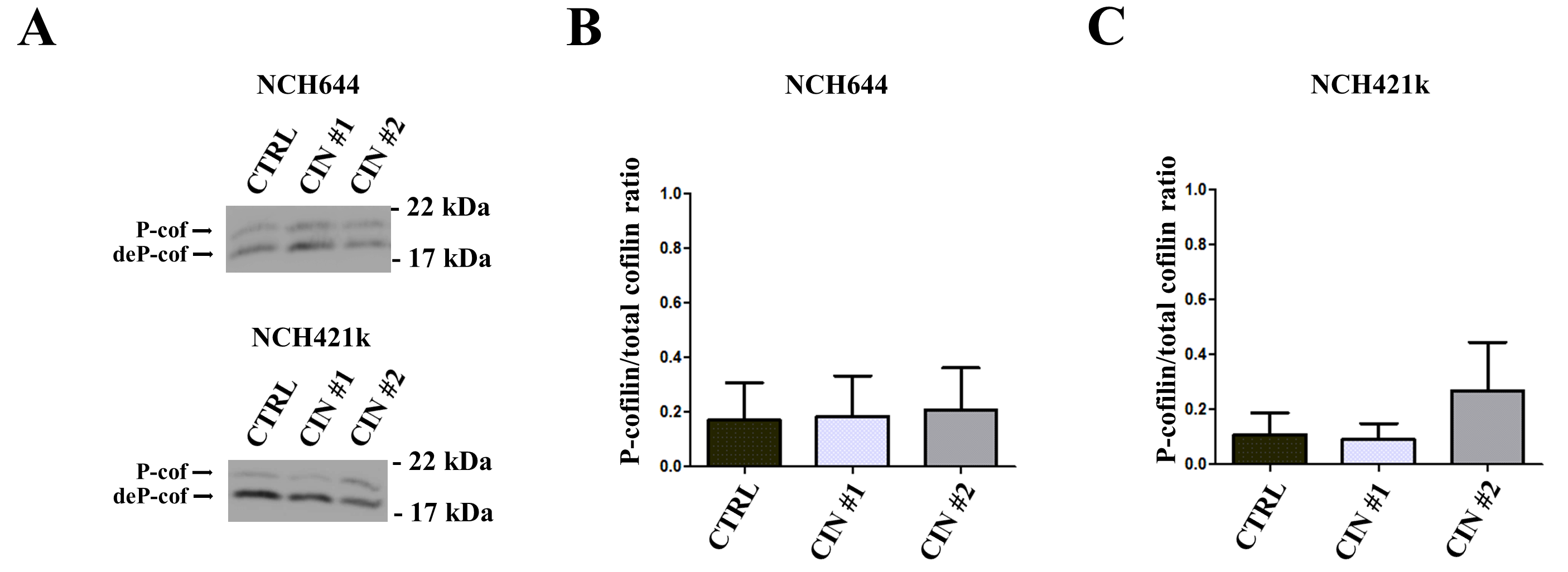

Supplement: Supplementary file 3 — Figure S2. Differentiation markers are induced upon CIN depletion. (A) and (B) Real-time PCR of the differentiation markers GFAP and TUBB3 in NCH644 (A) and NCH421k (B). There are significant differences between the groups for GFAP and TUBB3 in NCH644 as well as for GFAP in NCH421k (one-way anova, p < 0.05 for all comparisons mentioned, n = 2). (TIF 294 kb) [file 12885_2018_4440_MOESM3_ESM.tif]

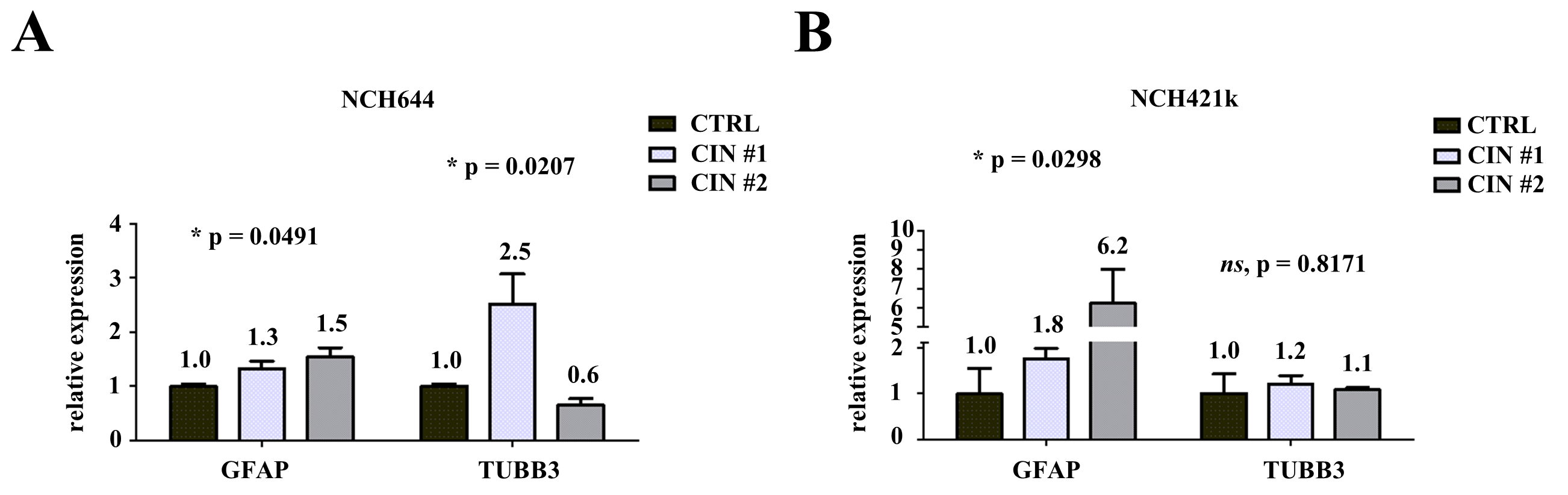

Supplement: Supplementary file 4 — Figure S3. Analysis of phosphocofilin levels in CIN knockdown cell lines. (A) Representative example of a western blot after PhosTag gel electrophoresis probed with α-cofilin antibody. (B) and (C) Quantification of western blots as shown in (A) for NCH644 (B) and NCH421k (C). There is no significant difference in the P-cofilin/total cofilin ratio between CTRL or CIN shRNA cells (n = 3, one-way ANOVA followed by Dunnett’s multiple comparisons test, p > 0.05). Shown are means + SD. (TIF 328 kb) [file 12885_2018_4440_MOESM4_ESM.tif]

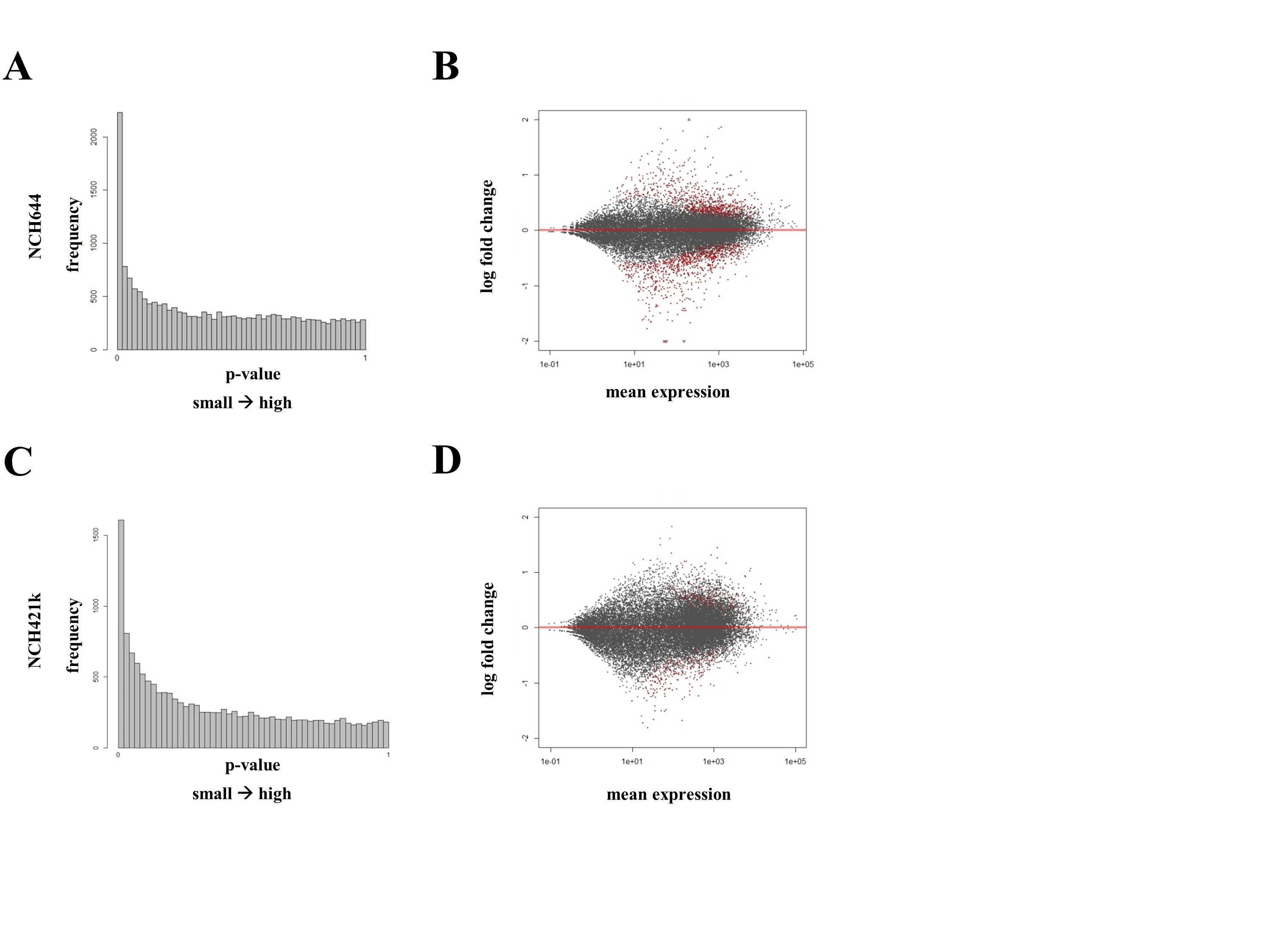

Supplement: Supplementary file 5 — Figure S4. Quality control plots for next generation sequencing data. (A) and (C) There is a high enrichment of small p-values after standard filtering in both NCH644 (A) and NCH421k (C) as calculated by DESeq2. (B) and (D) MA-plots indicate the presence of many deregulated genes (red) in both NCH644 (B) and NCH421k (D). (TIF 1008 kb) [file 12885_2018_4440_MOESM5_ESM.tif]

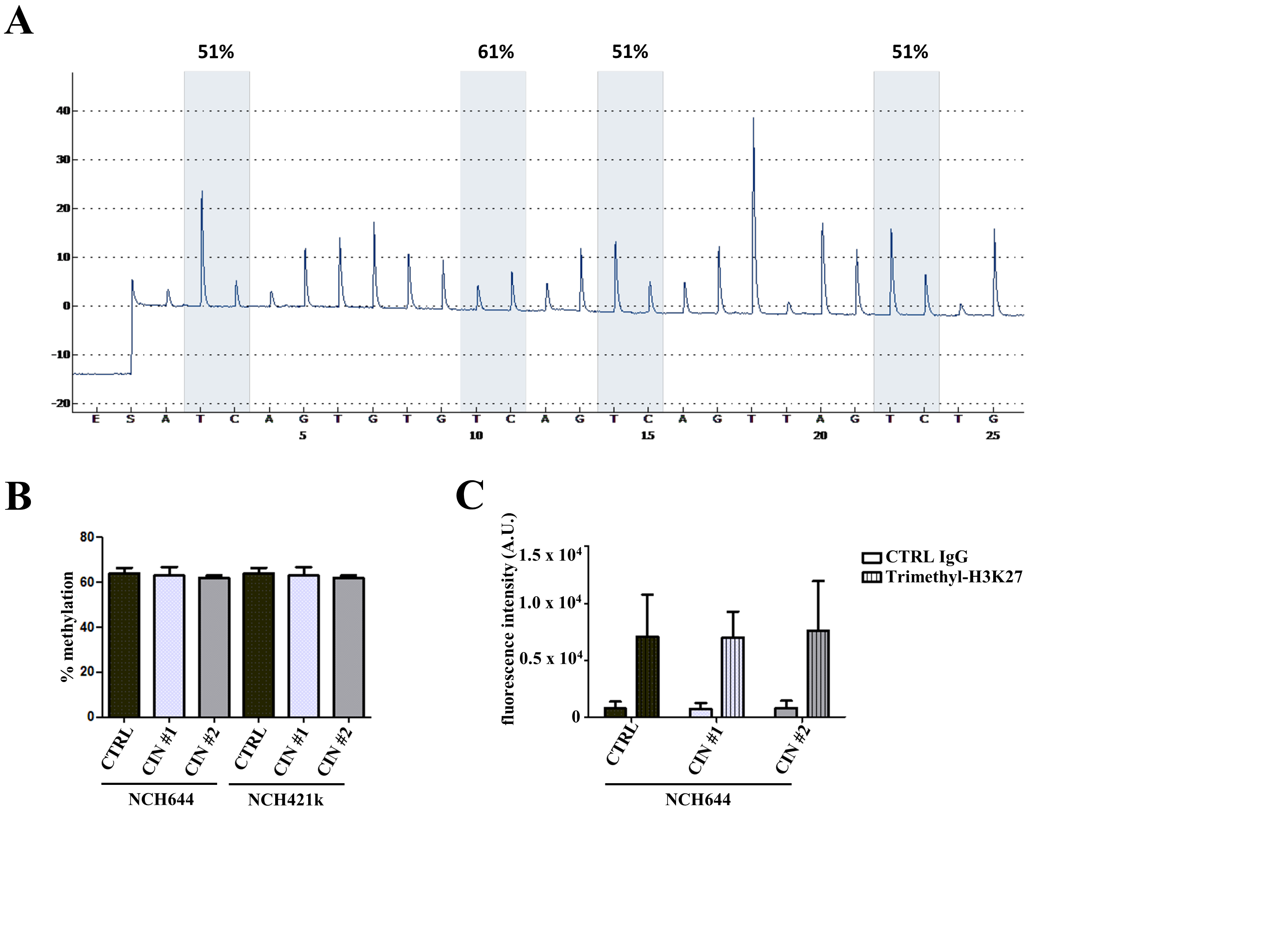

Supplement: Supplementary file 8 — Figure S5. Flow cytometry-based analysis of histone methylation levels and pyrosequencing-based analysis of global DNA methylation. (A) Exemplary sequencing run of a LINE-1 sequence from the Pyromark instrument. (B) Quantification of global DNA methylation profiles by LINE-1 element pyrosequencing in NCH644 and NCH421k (n = 3, one-way ANOVA followed by Dunnett’s multiple comparisons test, p > 0.05). Shown are means + SD. (C) Quantification of flow cytometry experiments of NCH644 cells stained with H3K27me3 or control IgG. Levels of histone H3 trimethylated at lysine 27 remain unchanged after CIN knockdown (n = 3, one-way ANOVA followed by Dunnett’s multiple comparisons test, p > 0.05). Shown are geometric means + SD. (TIF 893 kb) [file 12885_2018_4440_MOESM8_ESM.tif]

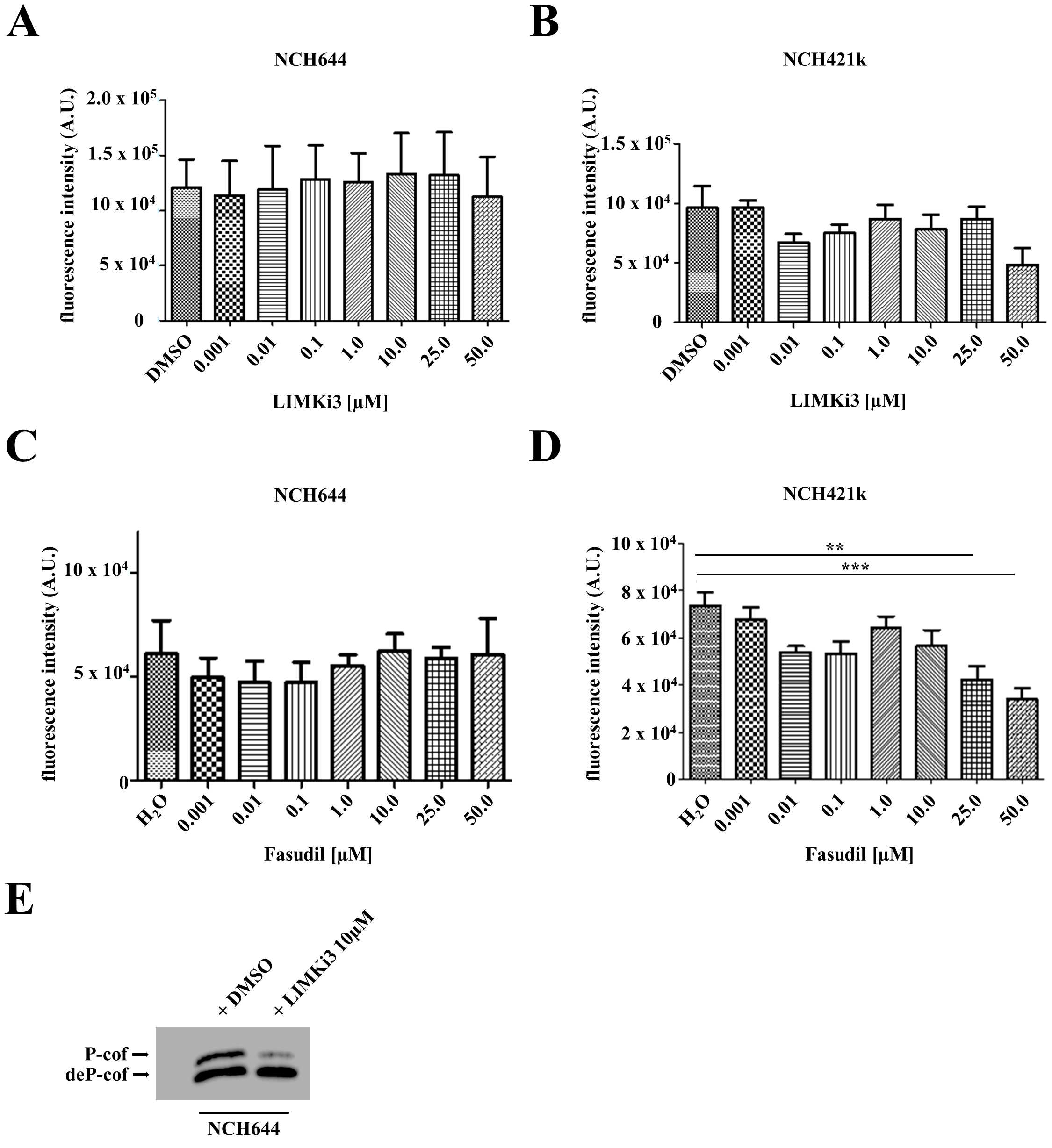

Supplement: Supplementary file 9 — Figure S6. Analysis of cell viability after LIMKi and fasudil treatment. (A) and (B) Measurement of cell viability with resazurin in NCH644 (A) and NCH421k (B) after LIMKi3 inhibitor treatment for 72 h. There is no significant decrease in cell viability after LIMKi3 application even at concentrations up to 50 μM (n = 3, one-way ANOVA followed by Dunnett’s multiple comparisons test, p > 0.05). Shown are means + SEM. (C) and (D) Measurement of cell viability with resazurin in NCH644 (C) and NCH421k (D) after fasudil treatment. A significant decrease in cell viability occurred only in NCH421k at the highest concentrations of 25 and 50 μM (n = 3, one-way ANOVA followed by Dunnett’s multiple comparisons test, p < 0.01 and 0.001, respectively). Shown are means + SEM. (E) Western blot after PhosTag gel electrophoresis probed with α-cofilin antibody. Inhibitor treatment with LIMKi3 strongly reduces the levels of p-cofilin. (TIF 1192 kb) [file 12885_2018_4440_MOESM9_ESM.tif]

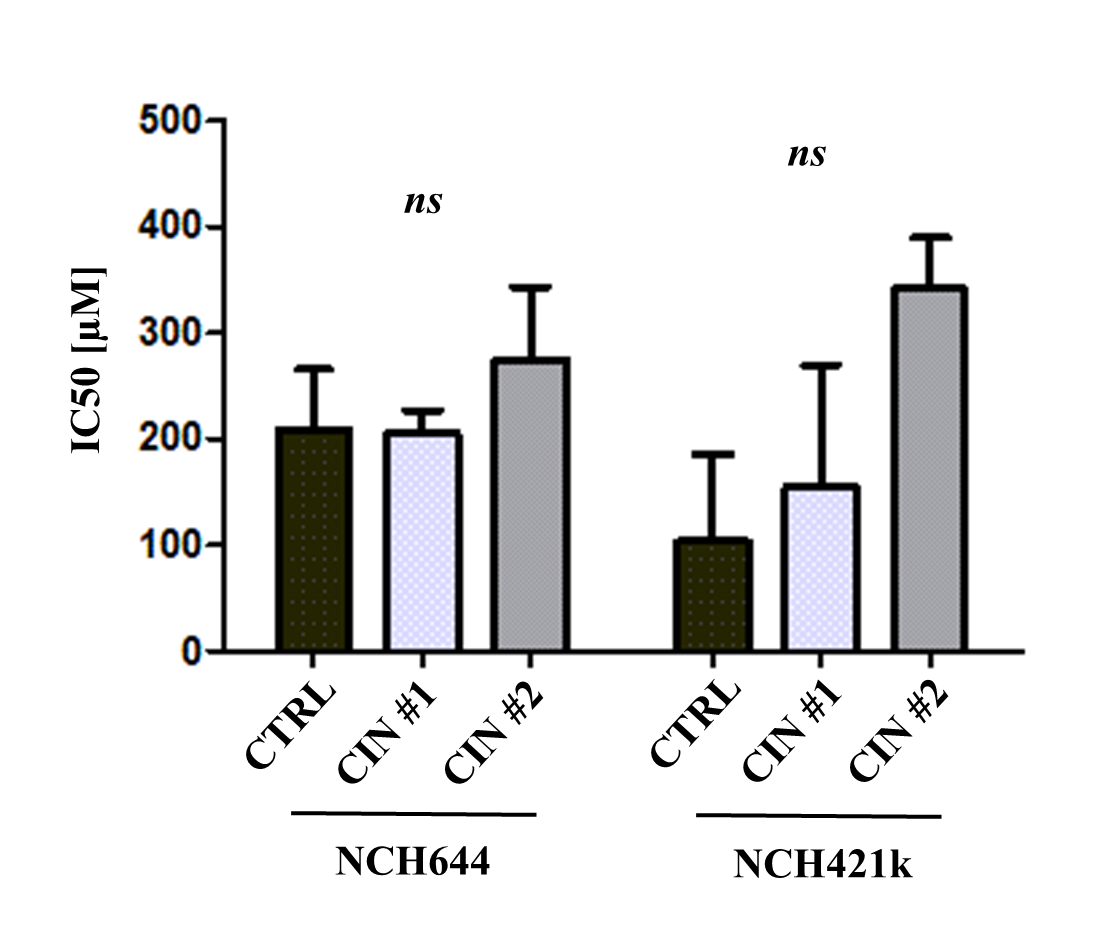

Supplement: Supplementary file 10 — Figure S7. Analysis of chemosensitivity in CIN knockdown cell lines. Temozolomide chemosensitivity remains unaltered after CIN knockdown. In both NCH644 and NCH421k there is no significant difference in IC50 values after CIN knockdown (n = 3, one-way ANOVA followed by Dunnett’s multiple comparisons test, p > 0.05). Shown are means + SD. (TIF 256 kb) [file 12885_2018_4440_MOESM10_ESM.tif]
